# Identification of novel potential drugs and miRNAs biomarkers in lung cancer based on gene co-expression network analysis

**DOI:** 10.5808/gi.23039

**Published:** 2023-09-27

**Authors:** Sara Hajipour, Sayed Mostafa Hosseini, Shiva Irani, Mahmood Tavallaie

**Affiliations:** 1 Biology Department, Science and Research Branch, Islamic Azad University, Tehran 14155-4933, Iran; 2 Chemical Injuries Research Center, Systems Biology and Poisonings Institute, Baqiyatallah University of Medical Sciences, Tehran 14359-16471, Iran; 3 Human Genetic Research Center, Baqiyatallah University of Medical Sciences, Tehran 14359-16471, Iran

**Keywords:** bioinformatics analysis, biomarker, drug-gene interaction, gene expression network, microRNA, non-small cell lung carcinoma

## Abstract

Non–small cell lung cancer (NSCLC) is an important cause of cancer-associated deaths worldwide. Therefore, the exact molecular mechanisms of NSCLC are unidentified. The present investigation aims to identify the miRNAs with predictive value in NSCLC. The two datasets were downloaded from the Gene Expression Omnibus (GEO) database. Differentially expressed miRNAs (DEmiRNA) and mRNAs (DEmRNA) were selected from the normalized data. Next, miRNA-mRNA interactions were determined. Then, co-expression network analysis was completed using the WGCNA package in R software. The co-expression network between DEmiRNAs and DEmRNAs was calculated to prioritize the miRNAs. Next, the enrichment analysis was performed for DEmiRNA and DEmRNA. Finally, the drug-gene interaction network was constructed by importing the gene list to dgidb database. A total of 3,033 differentially expressed genes and 58 DEmiRNA were recognized from two datasets. The co-expression network analysis was utilized to build a gene co-expression network. Next, four modules were selected based on the Z_summary_ score. In the next step, a bipartite miRNA-gene network was constructed and hub miRNAs (let-7a-2-3p, let-7d-5p, let-7b-5p, let-7a-5p, and let-7b-3p) were selected. Finally, a drug-gene network was constructed while SUNITINIB, MEDROXYPROGESTERONE ACETATE, DOFETILIDE, HALOPERIDOL, and CALCITRIOL drugs were recognized as a beneficial drug in NSCLC. The hub miRNAs and repurposed drugs may act a vital role in NSCLC progression and treatment, respectively; however, these results must validate in further clinical and experimental assessments.

## Introduction

Lung cancer is one of the main causes of the dead worldwild in recent decades and has become a communal healthiness subject [1]. Mostly, non–small cell lung cancer (NSCLC) causes the dominant population of lung malignancy cases. Among them, just 15% of cases have had a chance to be alive until the 5 years [2]. Mostly these cases have been recognized with metastatic or locally advanced disease while the prognoses are still insignificant [3]. Thus, there is a great significance to dedicating the predictive or diagnostic and molecular mechanisms and biomarkers for NSCLC pathogenesis.

The investigation has illustrated momentous relations among gene modifications, tumorigenesis and cancer progress of various kinds of NSCLC-relative tumors [4]. In current studies, the overexpression of CD44 was reported due to its crucial role in the occurrence development and migration of NSCLC [5]. Another study introduced some tumor-specifically methylated genes such as sperm associated antigen 6 which involved in NSCLC [6]. Lately, it was described that FPR1 mRNA levels in total plasma can prognoses small cell lung cancer (SCLC) and NSCLC [7]. Based on our previous knowledge, an enormous collection of non-coding RNAs are microRNAs that are 20–24 nucleotides in length on average. These miRNAs are elaborate in the fine-tuning of many biological processes. In a way that, they can bind to numerous target mRNAs typically and control the post-transcriptional of gene expression level [8]. Recent studies reported some miRNAs such as hsa-miR-30d [9] and miR-598 that have a possible tumor repressor role in the progression of NSCLC by repressing the migration and invasion in NSCLC [10]. However, the role of some miRNAs and genes there are still unknown in NSCLCs [11].

Many studies have used different bioinformatics analyses to identify novel and potential biomarkers that can detect cancers in the early stages [12-14]. For instance, Abedi et al. [15], used interactions among miRNAs and genes through bipartite networks and introduced miRNA and genes biomarkers in altered bladder cancer samples. Characterization of expression with high-throughput microarrays has become a major and common skill to obtain worldwide complementary insights into tumor genes and to identify new cancer biomarkers [11].

In the present evaluation, we presented a comprehensive analysis of miRNAs and gene expression by reanalyzing the GSE102286 and GSE101929 public datasets. The differentially expressed genes (DEGs) and miRNAs (DEmiR) were recognized in NSCLC models compared to healthy ones. Then, the DEmiRs-DEGs interactions were performed with subsequent analysis of functional enrichment to construct the regulatory networks of miRNA and genes. Eventually, a drug-gene interaction network was built using the DGIdb to detect candidate medicines to target the genes of hub miRNAs [16]. Through extensive bioinformatics investigation, we expect to discover new helpful goals and biomarkers for NSCLC.

## Methods

### Dataset and preprocessing

In the current evaluation, the expression data with GSE101929 and GSE102286 accession numbers were obtained from the Gene Expression Omnibus (GEO) database (https://www.ncbi.nlm.nih.gov/geo/). The GSE101929 and GSE102286 have 66 (containing 34 normal and 32 NSCLC samples) and 179 (containing 88 normal and 91 NSCLC samples) samples, respectively. These datasets were downloaded using the GEOquery package [17]. Then, the data were normalized with log2 +1 and normalized quantile method [18], and DEGs were extracted using R, limma package [19]. Next, up and down regulated gene and miRNA lists were achieved.

### Co-expression network analysis

To build the co-expression network, DEGs were utilized. Next, we constructed a co-expression network using the WGCNA package [20]. A β parameter (soft threshold power beta) was utilized to arrange the scale-free property of the network, and then arranged for 7. Scale independence and mean connectivity of R^2^ and various soft thresholds are indicated in (Supplementary Fig. 1. Among powers alternating from 1 to 20, β = 7 was chosen to find scale independence for the network and the scale free exponent R^2^ was 0.952. A topological overlap matrix was computed afterward creating the adjacency matrix of the expression data. The WGCNA classified grouping was applied to elicit the modules of the co-expression network. Additionally, the *DeepSplit* and Minimum Module Size limitations were allocated values of 4 and 20, respectively, by looking at various parameters. The elicited modules were then combined and color-labeled.

The Z_summary_ value was employed to dissect module preservatione. Z_summary_ score is imputed from half of the sum of Z_density_ and Z_connectivity_. The Z_summary_ score was utilized to compare the significance of detected statistics. The Z_summary_ values were considered ≤2, between 2 and 10, and >10 as not conserved, temperately conserved, and powerfully conserved, separately [21]. The modules that have Z_summary_ values of more than 10 did not offer any details because they were powerfully conserved; therefore, these modules were not utilized (Supplementary Fig. 2).

### miRNA-mRNA bipartite network reconstruction

First, we extracted miRNAs that target the genes in all selected modules from the miRWalk 2.0 database. Considering that miRNA-mRNA interactions have been confirmed experimentally, we constructed a bipartite network using miRNAs and genes derived from selected modules. It is noticeable that we used the DEmiRNAs at the final to construct a bipartite network containing 119 genes and 29 miRNAs. Next, by studying the relationships between genes and their cooperating miRNAs, the node miRNAs with the maximum connectivity were taken to continue substantial contacts and elude density. Finally, the output file was imported to Cytoscape [22] software to visualize a further analysis.

### Enrichment analysis of DEgenes and DEmiRNAs

Functional enrichment analysis was implemented via the DAVID [23] database (https://david.ncifcrf.gov/) for identifying the most related biological mechanisms to the genes. This database was used for annotation, visualization, and Integrated discovery. Moreover, Reactome [24] pathway database was utilized to identify the pathway enrichment analysis. In the next step, the TAM tool [25] was utilized to enrich the miRNAs. Furthermore, the function and family of the miRNAs were identified using the TAM tool by defaulting limitations.

### Construction of drug-gene interaction network

The DGIdb (https://www.dgidb.org/) (Drug Gene Interaction Database) [26] was utilized for detecting the candidate drugs that target the genes. This database is associated with 22 other connected databases that obtain the interaction based on 24 associated databases. In this study, only approved interactions were used to identify drug–gene interactions information. After importing the list of genes to the DGIdb database, the list of drug-gene interactions was obtained.

## Results

### Identification of differentially expressed genes and miRNAs

To find a list of genes and miRNAs that are involved in NSCLC, we evaluated GSE101929 and GSE102286 datasets. Table 1 provides more information about these datasets. Finally, 3033 and 58 DEGs and miRNAs were obtained, respectively (Supplementary Tables 1 and 2) while 1,244 genes were meaningfully up-regulated, 1,790 genes were significantly down-regulated (Supplementary Table 1). Also, 47 miRNAs were significantly up-regulated and 11 miRNAs were significantly down-regulated (Supplementary Table 2).

### Module detection and co-expression network analysis

Three gene co-expression network was built through WGCNA on 3,033 DEGs. In this network, the lowest and the biggest modules were plum1 and yellow including 21 and 204 genes, individually. The grey module in this network incorporated 108 genes, which were omitted from the additional investigation. The Supplementary Table 3 indicated more information about all modules. Additionally, cluster dendrogram are shown in Supplementary Figs. 3 and 4. The unpreserved modules might modify numerous signaling cascades and cause progression cancer. After Z_summary_ values imputing for all modules, the thresholds = 2.5 were used for module (Supplementary Fig. 1). Thus, modules with a Z_summary_ ranging from 1 to 2.5 were chosen as important modules. These modules are displayed as unpreserved and could be valuable in the development of NSCLC. Lastly, the type of network was selected as the signed-hybrid network. The conservation of median rank and Z_summary_ beside the module size is illustrated in Supplementary Fig. 2. Finally, 4 modules with Z_summary_ scores ≤2.5 were chosen (Table 2 illustrates certain modules by their characteristics). Consequently, yellow green, light green, plum1, and sky blue modules revealed low conservation (Table 1).

### miRNA-mRNA bipartite network reconstruction

**Fig. 1.**
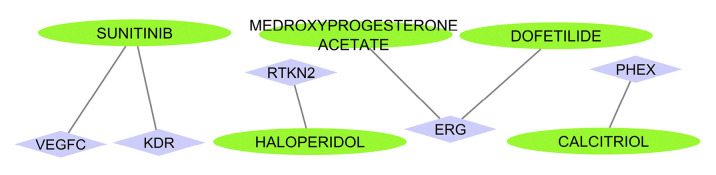
Bipartite miRNA-mRNA subnetwork. The Cytoscape v.3.8.2 was utilized to imagine the network.

A miRNA-mRNA bipartite network was assembled using selected modules and their relevant miRNAs. The microRNAs with more connections controlled an additional important number of genes, utilizing a significant influence on the post-transcriptional rule. Next, the top 5 miRNAs (hubs) were selected from the network and utilized for extra investigation besides their target genes. It is vital to choose one hub miRNAs to elude difficulty and eliminate miRNAs with small properties on the network and pathogenesis. Fig. 1 indicates the bipartite network with five hub miRNAs and related genes.

### Enrichment analysis of miRNAs and genes

In this section, the gene ontology enrichment analysis was performed to evaluate the significant pathways, and the Reactome pathway database was utilized for the path enrichment investigation. The result indicated the most important pathways for genes in bipartite network were “Tight junction,” “Epithelial cell signaling in *Helicobacter pylori* infection,” and “Focal adhesion.” Also, the TAM tool was used to annotate hub miRNAs. The most important functions for hub miRNAs were “Cell deviation,” “Folliculogenesis,” “Adipocyte differentiation,” “T-cell differentiation,” “Hematopoiesis,” “Aging,” “Tumor suppressor miRNAs,” “Cell death,” “Glucose metabolism,” “Innate immunity,” “Cell cycle,” “Cell proliferation,” and “Bone regeneration.”

### Construction of drug-gene interaction network

**Fig. 2.**
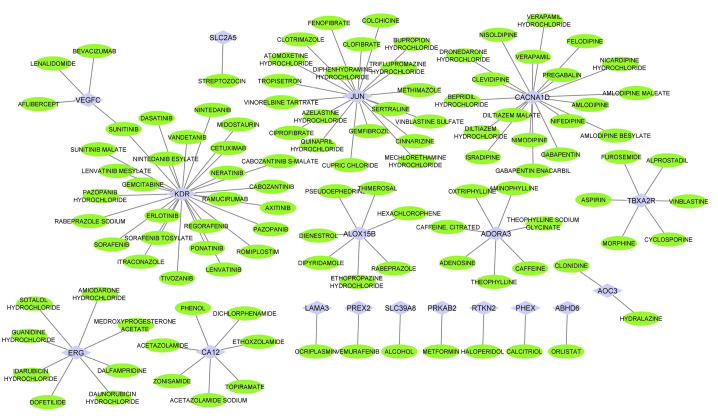
The candidacy drug-gene network extracted from DGIdb database. The candidacy drugs (green elips) recognized as controllers of the genes. Cytoscape v.3.8.2 was utilized to imagine the network.

**Fig. 3.**
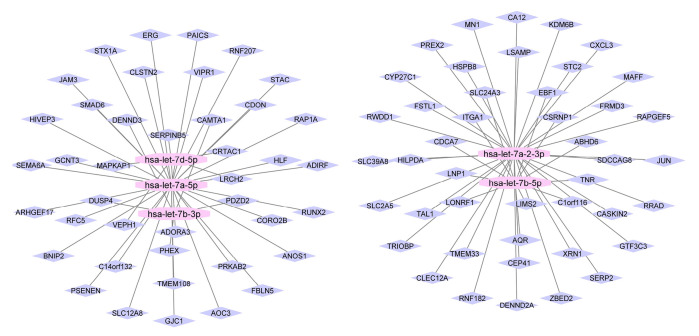
Five important drugs with their targets.

The DGIdb was utilized to find drugs that target the genes. After importing the genes into DGIdb, drug-gene interactions were collected for the genes by restrictive medicines to agreed medicines. Then, the drug-gene interactions network is constructed and visualized by Cytoscape (version 3.8.2). As a result, there were a lot of drugs in this network (Fig. 2). Noticeably, the drug-gene network, SUNITINIB targeted vascular endothelial growth factor C (VEGFC) and KDR (Fig. 3). After analyzing the network, based on hub degree, the important drugs were extracted. Fig. 3 provides the five important drugs including SUNITINIB, MEDROXYPROGESTERONE ACETATE, DOFETILIDE, HALOPERIDOL, and CALCITRIOL with their targeted genes.

## Discussion

In the current evaluation, the expression profile of miRNAs and miRNAs was evaluated in NSCLC to determine miRNA biomarkers, and the co-expression network analysis was completed based on gene expressions. First, differentially expressed and significant mRNAs (adjusted p-value < 0.01 and log FC > |1|) and miRNAs (adjusted p-value < 0.01) were chosen. Therefore, 3033 genes were utilized to build the co-expression network. Next, a co-expression network was reconstructed using the WGCNA package. The extracted modules were merged based on Z_summary_. Meanwhile, as the distinction of very connected modules is problematic, it is suggested to combine them [27]. Then, a bipartite network was built with miRNAs and their cooperating genes. Hub miRNAs with an essential character in the gene modulation were identified and utilized to generate a subnetwork. Subsequently, a drug-gene interaction network was constructed and important drugs were repurposed by importing the genes in the bipartite network. The mentioned miRNAs were the future biomarkers of NSCLC and were found via our method. We studied the found biomarkers exactly.

In the first step, the miRNAs we studied in the literature [15]. All miRNAs that were obtained belong to the let-7 family. It is reported that miRNA-let-7a (let-7a) can suppress cell development in numerous cancers, and it is downregulated in lung adenocarcinoma tissues compared with regular tissues [28]. Also, let-7a overexpression successfully repressed cancer migration, invasion, and cell proliferation in A549 and H1299 cells while it stimulated cell apoptosis and cell cycle arrest. Additionally, regulating cyclin D1 signals are performed by let-7a and lead to reduce cell growth [28]. Increasing lung tumor development was reported by losing the let-7 role in mouse models, while let-7 exogenous transfer decreased the tumor weight by recognizing cancers in mouse replicas of NSCLC [29]. In another study let-7g efficiently encourages cell cycle detention and cell death in K-RasG12D expressing murine lung cancer cells by targeting *KRAS* oncogene. Certainly, in lung adenocarcinoma (LUAD) cells and tissue samples let-7b-3p was down-regulated that is in relation with poor prognoses in LUAD patients while targeted the BRF2-mediated MAPK/ERK pathway. So, it can suppress the LUAD cells proliferation and metastasis *in vivo* and *in vitro* [30]. These indications reveal that the let-7 family can help as a predictive sign and beneficial aim in NSCLC [31]. Based on previous investigations, the let-7 family is well known as lung cancer associated miRNA that is in line with the candidate miRNAs that were found in our projected method. The role of our finding miRNAs did not report exactly in NSCLC [32]. We advise that these miRNAs might be potentially linked with NSCLC and can be important biomarkers for NSCLC diagnosis in the initial identification of NSCLC.

Among other datasets from GEO, the datasets were selected with some options for focusing and exploring on the relationships between miRNA and mRNA, specially, based on our project designing. Finally, and it clearly was beneficial when it validated our interactions. By using these purpose-driven datasets, we made sure that our suggested miRNA-mRNA associations had undergone rigorous experimental validation, which boosts our confidence in the results. Moreover, the careful selection of these datasets allowed us to delve deeply into these interactions and uncover networks that govern a wide range of biological processes [32]. The specific and discovered miRNA-mRNA associations in this study are potential as promising candidates for experimental validation and functional studies by providing fresh insights into biological mechanisms [33]. However, in this study there were some limitations in terms of generalizability to all gene interactions. Thus, the conclusions drawn from the two datasets may not fully represent the spectrum of miRNA-mRNA regulatory interactions. To address this limitation, future research should consider incorporating datasets from experimental conditions and biological contexts to enhance our understanding of miRNA-mRNA networks [16].

In the subsequent section, important drugs and their target genes were evaluated. Sunitinib targeted VEGFC and KDR genes by inhibiting the tyrosine kinase receptors, containing platelet-derived development factor receptors and vascular endothelial development factor receptors, which have single-agent antitumor action in intractable NSCLC [34]. It has been reported that patients with progressive lung adenocarcinoma preserved with medroxyprogesterone, celecoxib, and dietary interventions may experience important improvement in some Systemic Immune-Metabolic Syndrome outcomes [35]. The mechanism of medroxyprogesterone is unclear in cancer but it may be associated with appetite stimulation [35]. Haloperidol was an important drug in our result and this drug was repurposed by evaluating drug repositioning NSCLC using gene co-expression and drug-gene interaction networks analysis [36]. There is not any evidence about the relationship between NSCLC and other drugs like calcitriol and dofetilide. Based on investigation in literature, some of our extracted drugs were approved to treat NSCLC. However, some of them have not been evaluated in clinical investigates that can be studied experimentally.

Our study was designed to present drug candidates and potential miRNAs as a biomarkers in NSCLC by performing a predictive method. The gene co-expression network analysis process was applied to the data obtained from the GEO database. Then, bipartite networks (miRNA-mRNA) were obtained from the co-expression networks which were reconstructed on the genes from significant modules. Subsequently, hub miRNAs were also identified. These miRNAs that have the maximum degree of connectivity were considered as probable predictive biomarkers for NSCLC. Based on our main aim, these miRNAs were found to target modulus genes while there is not any information on their expression level in NSCLC. Therefore, their expression level assessment would be essential for the validity of NSCLC in future experimental studies like clinical trials. Various therapies such as immunotherapy, targeted therapy and chemotherapy have been approved to improve survival in patients with advanced and metastatic cancers. However, genomic unpredictability and signal transduction redundancy are challenges in the NSCLC treatment. Planning is needed to increase the efficacy of drug interactions in the preclinical setting. The current complementary assessment emphasized the status of this topic in the provision of medicine. Furthermore, this study presented a powerful tool for developing approaches to the discovery and development of new drugs.

## Figures and Tables

**Table 1.** Number of data samples according to their sexes in GSE101929

Sex	Normal	Tumor
Male	15	15
Female	19	17
Total	32	32

**Table 2.** Extracted modules and their properties

	Module color	Value of Z_summary_	No. of genes in module
1	Yellow green	1.5	24
2	Light green	2.2	66
3	Blue sky	2.0	24
4	Plum1	1.8	21

## Supplementary Materials

Supplementary Table 1. Differentially expressed genes

Supplementary Table 2. Differentially expressed miRNA

Supplementary Table 3. More information about all modules

Supplementary Fig. 1. Scale independence and mean connectivity of R2 and various soft thresholds.

Supplementary Fig. 2. Z_summary_ score.

Supplementary Fig. 3. Dendrogram of modules.

Supplementary Fig. 4. Dendrogram after merging modules.
