# Supplementary material for: Identification of novel potential drugs and miRNAs biomarkers in lung cancer based on gene co-expression network analysis
Source: Genomics Inform. 2023 Sep 27;21(3):e38. doi: 10.5808/gi.23039 (PMC10584645; doi:10.5808/gi.23039)
Supplement: Supplementary Table 1. — Differentially expressed genes [file gi-23039-Supplementary-Table-1.pdf]

Supplementary Table 1. Differentially expressed genes

Gene.symb	adj.P.Val	p-value
SLC6A4	2.77E-21	7.10E-25
CLDN18	6.93E-18	1.09E-20
AGER	3.33E-21	9.75E-25
SCGB1A1	2.23E-08	9.88E-10
CA4	1.35E-28	2.46E-33
TMEM100	7.22E-18	1.20E-20
FCN3	1.91E-16	6.24E-19
FAM107A	5.85E-20	2.99E-23
SOSTDC1	5.42E-18	7.83E-21
SFTPC	2.13E-11	3.61E-13
GPM6A	8.82E-22	2.10E-25
GDF10	9.87E-23	1.48E-26
ADRB1	1.74E-15	8.39E-18
ADAMTS8	3.08E-20	1.41E-23
PIR-FIGF	1.91E-13	1.70E-15
TNNC1	1.41E-18	1.45E-21
GKN2	1.03E-12	1.18E-14
CD300LG	5.41E-16	2.05E-18
GPIHBP1	1.50E-15	6.99E-18
LRRN3	1.03E-17	1.90E-20
CSF3	9.33E-10	2.71E-11
CPB2	1.29E-14	7.95E-17
MAMDC2	1.14E-13	9.54E-16
GRIA1	9.87E-23	1.29E-26
FGFR4	7.22E-18	1.20E-20
FABP4	2.13E-13	1.91E-15
CYP4B1	2.71E-11	4.77E-13
TNXB	4.08E-14	2.92E-16
TPPP3	3.04E-12	3.99E-14
C2orf40	4.17E-11	7.69E-13
STXBP6	1.32E-18	1.28E-21
EXOSC7	1.73E-15	8.30E-18
PGC	7.26E-08	3.82E-09
BTNL9	8.86E-17	2.48E-19
SUSD2	2.92E-11	5.15E-13
FXYD1	2.90E-14	2.01E-16
SOX7	9.92E-13	1.13E-14
NTRK2	9.63E-12	1.45E-13
MCEMP1	2.86E-10	6.94E-12
WIF1	1.06E-09	3.15E-11
NCKAP5	3.22E-21	8.83E-25

FHL1	1.02E-19	6.33E-23
ACADL	2.38E-17	5.49E-20
IGSF10	6.64E-19	5.71E-22
MT1M	1.46E-10	3.21E-12
VIPR1	2.88E-15	1.49E-17
SERTM1	2.28E-19	1.58E-22
INMT	5.71E-14	4.33E-16
KCNK3	2.88E-15	1.50E-17
GPER1	3.25E-14	2.28E-16
IL1RL1	2.85E-16	9.87E-19
SEMA3G	1.32E-15	5.95E-18
FAM150B	6.42E-13	6.83E-15
ADAMTSL3	5.66E-16	2.16E-18
CLIC5	1.78E-17	3.77E-20
SGCG	1.19E-20	4.34E-24
FAT3	1.67E-16	5.29E-19
SCN4B	7.69E-16	3.14E-18
MYZAP	2.90E-19	2.12E-22
ADIRF	1.23E-12	1.45E-14
TCF21	1.63E-17	3.40E-20
MASP1	7.86E-20	4.46E-23
TCEAL2	1.25E-18	1.19E-21
HIGD1B	5.15E-15	2.86E-17
SCN7A	6.98E-18	1.12E-20
SDPR	6.54E-19	5.50E-22
BCHE	5.30E-12	7.56E-14
LINC00968	1.70E-16	5.40E-19
LRRC36	3.16E-12	4.16E-14
NDRG4	1.59E-11	2.56E-13
PEBP4	6.80E-08	3.54E-09
SCARA5	3.45E-12	4.61E-14
PLAC9	1.64E-16	5.09E-19
VEPH1	7.12E-10	1.99E-11
RXFP1	4.54E-23	3.32E-27
ABCA8	5.15E-13	5.23E-15
EMCN	2.58E-16	8.83E-19
TBX2	2.68E-15	1.36E-17
FHL5	7.72E-19	6.77E-22
SH3GL3	3.05E-26	1.67E-30
DNASE1L3	1.08E-10	2.27E-12
SHROOM4	5.87E-18	8.69E-21
C9orf24	1.64E-05	1.84E-06
TAL1	5.62E-19	4.53E-22

LAMC3	2.45E-12	3.13E-14
LIMS2	2.79E-17	6.68E-20
RAMP3	6.48E-16	2.54E-18
RTKN2	6.54E-15	3.74E-17
KANK3	3.92E-19	2.94E-22
TMSB15B	1.10E-13	9.07E-16
TMEM88	1.06E-15	4.63E-18
FENDRR	2.32E-18	2.59E-21
PPBP	3.23E-08	1.51E-09
HSPB8	5.73E-14	4.36E-16
HYAL1	1.15E-08	4.68E-10
ANGPTL1	3.63E-18	4.31E-21
WFDC1	2.21E-14	1.47E-16
MFAP4	3.38E-11	6.08E-13
FAM189A2	5.89E-16	2.29E-18
ACVRL1	1.08E-20	3.55E-24
ADH1B	4.06E-16	1.48E-18
DEFA1B	7.78E-08	4.14E-09
RNF182	3.29E-17	8.13E-20
GPR146	1.67E-16	5.24E-19
ADAMTS9-A	9.78E-17	2.77E-19
FGFBP2	2.05E-15	1.02E-17
LYVE1	4.06E-11	7.48E-13
PTPRB	3.54E-20	1.69E-23
DUOX1	2.96E-09	9.99E-11
LOC101929	1.18E-17	2.27E-20
CCBE1	2.84E-19	2.03E-22
LMOD1	8.45E-10	2.42E-11
DACH1	3.20E-17	7.85E-20
PLA2G1B	7.80E-09	3.02E-10
TTN	3.51E-11	6.37E-13
DES	8.52E-13	9.51E-15
PCAT19	8.71E-18	1.50E-20
TPPP	2.88E-13	2.70E-15
UPK3B	2.02E-08	8.89E-10
TEK	9.32E-19	8.35E-22
GPC3	5.36E-11	1.02E-12
C1QTNF7	2.53E-15	1.27E-17
FOSB	5.65E-07	3.97E-08
ASPA	1.65E-11	2.68E-13
LOC101926	3.21E-09	1.09E-10
GFRA1	1.74E-10	3.95E-12
LDB3	1.18E-11	1.82E-13

ZBTB16	4.20E-08	2.05E-09
TUBB1	2.06E-14	1.36E-16
HHIP	4.22E-08	2.06E-09
S100A3	1.90E-15	9.31E-18
SFTPA2	7.37E-06	7.38E-07
KRT4	9.41E-10	2.74E-11
AFF3	1.28E-10	2.77E-12
CDH19	1.38E-17	2.80E-20
LOC101060	1.21E-13	1.03E-15
TBX4	5.90E-15	3.34E-17
RAMP2	1.52E-14	9.64E-17
PRX	1.23E-19	8.05E-23
SEMA6A	1.22E-12	1.44E-14
ANXA8L1	1.39E-08	5.75E-10
FAM46B	5.65E-16	2.15E-18
CCDC85A	2.07E-16	6.94E-19
HSPC324	1.07E-15	4.69E-18
SSTR1	2.70E-13	2.50E-15
FRAS1	1.36E-09	4.15E-11
TBX5-AS1	1.03E-12	1.18E-14
C20orf85	5.04E-05	6.63E-06
MIR3945HG	5.35E-10	1.42E-11
ITGA1	1.77E-16	5.67E-19
FLJ30901	1.33E-18	1.31E-21
MS4A15	4.37E-14	3.19E-16
MYRF	3.51E-16	1.25E-18
JAM2	2.91E-18	3.36E-21
AGTR1	2.61E-12	3.37E-14
FMO2	9.26E-17	2.61E-19
IRX2	1.55E-06	1.26E-07
NTNG1	2.69E-20	1.09E-23
GLDN	9.59E-09	3.80E-10
ERBB4	2.88E-13	2.70E-15
SH2D3C	1.22E-14	7.41E-17
CNKSR2	8.06E-16	3.33E-18
KLK10	1.08E-07	6.04E-09
NFASC	7.74E-13	8.47E-15
CYP3A5	2.07E-07	1.27E-08
SLC14A1	1.30E-13	1.12E-15
GPM6B	9.57E-18	1.72E-20
PPP1R14A	8.91E-11	1.82E-12
COL6A6	7.28E-16	2.94E-18
STARD9	1.36E-16	4.13E-19

KANK4	1.32E-10	2.87E-12
ICAM4	5.00E-09	1.81E-10
KL	9.82E-18	1.78E-20
HSPA12B	6.89E-17	1.88E-19
ANGPT1	1.74E-11	2.86E-13
ACKR4	2.45E-17	5.68E-20
CDO1	1.80E-13	1.59E-15
TRHDE	5.30E-12	7.55E-14
EGFL7	7.49E-11	1.51E-12
LRRC32	5.97E-13	6.21E-15
MARCO	4.44E-06	4.14E-07
EDN1	1.55E-13	1.36E-15
KLF4	6.24E-12	9.03E-14
HSD17B6	6.22E-09	2.32E-10
MMP28	4.52E-10	1.17E-11
EDNRB	3.54E-20	1.75E-23
ADRA1A	6.81E-16	2.70E-18
SNTN	8.57E-05	1.22E-05
GPD1	1.22E-12	1.43E-14
SYNM	2.36E-14	1.60E-16
ADGRB3	1.05E-16	3.05E-19
RASGRF1	3.73E-09	1.30E-10
CHRDL1	3.34E-10	8.28E-12
P3H2	2.63E-08	1.20E-09
CACNA1D	4.29E-11	7.94E-13
ATP1A2	4.95E-12	6.96E-14
MME	2.45E-16	8.30E-19
DYNLRB2	2.86E-05	3.46E-06
TSPAN7	4.15E-13	4.07E-15
FUT1	5.63E-11	1.09E-12
NOS1	1.33E-11	2.08E-13
ZNF385B	8.96E-07	6.74E-08
STARD13	3.95E-18	4.78E-21
ADRB2	8.04E-16	3.31E-18
SELE	0.000701	0.000136
ARHGAP6	5.31E-16	2.00E-18
TSPAN18	1.96E-12	2.45E-14
WISP2	6.92E-11	1.38E-12
SLIT3	4.29E-15	2.34E-17
COLGALT2	1.87E-16	6.04E-19
ANKRD29	5.05E-13	5.11E-15
MYH11	9.98E-11	2.08E-12
LEPROT	3.53E-17	8.98E-20

STX11	2.21E-13	2.00E-15
SEMA6D	8.26E-13	9.13E-15
RFX2	1.30E-08	5.30E-10
SFTPD	4.75E-05	6.20E-06
EFCC1	9.43E-16	4.04E-18
SPOCK2	1.15E-18	1.07E-21
OGN	2.26E-10	5.33E-12
HBG2	1.01E-08	4.00E-10
PKNOX2	7.25E-13	7.86E-15
AQP4	6.65E-08	3.45E-09
SFTA1P	3.55E-10	8.91E-12
CDH5	6.86E-17	1.86E-19
KCNJ15	3.82E-06	3.49E-07
HBB	1.10E-10	2.33E-12
PRKCE	1.23E-19	8.10E-23
CD36	6.20E-10	1.70E-11
SMAD6	8.28E-15	4.84E-17
NOSTRIN	1.13E-14	6.76E-17
FOXF1	9.99E-16	4.31E-18
RASIP1	6.93E-18	1.10E-20
SCGB3A2	0.000144	2.21E-05
TBX3	1.24E-14	7.55E-17
KLK11	3.18E-06	2.83E-07
RHOJ	7.27E-13	7.89E-15
SORBS2	1.93E-11	3.22E-13
S100A12	7.84E-06	7.93E-07
CACNA2D2	1.38E-07	8.00E-09
KLB	1.25E-09	3.81E-11
CREB5	6.45E-09	2.42E-10
CAV2	2.15E-18	2.32E-21
GHR	5.49E-13	5.64E-15
CX3CR1	4.20E-08	2.05E-09
SOX17	1.38E-13	1.20E-15
ITGA8	2.52E-12	3.24E-14
SCGB3A1	0.000907	0.000182
SLC46A2	1.91E-06	1.59E-07
LIN7A	3.46E-17	8.74E-20
NEGR1	1.32E-13	1.14E-15
SLC5A9	2.87E-13	2.69E-15
ABCA3	3.84E-06	3.50E-07
TGFBR3	6.45E-17	1.73E-19
LOC285043	2.37E-08	1.07E-09
CCM2L	2.95E-15	1.54E-17

DNAH12	4.67E-05	6.08E-06
C14orf132	3.33E-15	1.77E-17
ACKR1	1.08E-06	8.34E-08
CHI3L2	1.33E-07	7.65E-09
TXNL1	5.03E-18	7.18E-21
SYNE1	8.25E-15	4.81E-17
RBMS3	6.31E-18	9.58E-21
LINC00312	1.97E-14	1.29E-16
GRK5	1.08E-26	3.96E-31
NEBL	1.97E-17	4.25E-20
CAV1	4.20E-18	5.45E-21
HBA2	3.07E-10	7.53E-12
ROBO4	3.95E-16	1.44E-18
VSIG2	1.95E-10	4.49E-12
MYOCD	7.83E-10	2.22E-11
PDE1C	8.71E-18	1.49E-20
TMEM110-M	3.57E-10	8.97E-12
OR7E47P	1.38E-11	2.18E-13
CALCRL	3.91E-17	1.01E-19
CASQ2	3.47E-09	1.20E-10
TMEM108	9.45E-13	1.07E-14
AOC3	6.11E-13	6.39E-15
PGM5	3.96E-12	5.36E-14
LOC100996	4.08E-13	3.98E-15
ROBO2	3.31E-14	2.33E-16
RETN	1.83E-07	1.10E-08
PCDH9	3.96E-12	5.36E-14
SLIT2	1.28E-15	5.74E-18
CORO2B	3.18E-15	1.67E-17
PGR	2.02E-11	3.40E-13
RGN	1.11E-10	2.35E-12
ERICH3	1.19E-05	1.28E-06
DENND2A	3.05E-22	5.60E-26
TMEM190	0.000295	5.02E-05
RAI2	2.57E-15	1.30E-17
CLDN5	6.97E-16	2.79E-18
FAM167A	8.10E-12	1.19E-13
PDE8B	1.49E-17	3.08E-20
KIAA1462	7.18E-17	1.97E-19
AGTR2	1.41E-05	1.54E-06
TMEM178A	2.96E-10	7.23E-12
TNR	1.04E-07	5.79E-09
FLJ35700	1.57E-06	1.28E-07

LRRN4	1.09E-07	6.09E-09
C1orf168	3.15E-08	1.47E-09
HECW2	1.09E-12	1.26E-14
SEMA5A	5.56E-14	4.19E-16
ZBED2	4.27E-06	3.96E-07
LTBP4	1.98E-13	1.77E-15
NPR1	7.22E-20	3.96E-23
HPGDS	1.36E-06	1.08E-07
SUPT20H	1.08E-12	1.24E-14
ADGRL2	5.85E-16	2.26E-18
PHEX	5.64E-09	2.08E-10
NAV2	6.84E-13	7.34E-15
ST6GALNAC	2.49E-12	3.19E-14
RSPO2	5.29E-14	3.95E-16
MIR6872	3.94E-12	5.32E-14
CRYAB	1.34E-11	2.10E-13
FIBIN	1.61E-09	5.01E-11
EML1	1.55E-16	4.78E-19
GNG11	1.39E-15	6.36E-18
DKK2	5.60E-10	1.50E-11
ID4	3.09E-10	7.59E-12
LDB2	2.21E-17	4.98E-20
CYS1	2.38E-14	1.61E-16
ITIH5	2.80E-15	1.43E-17
EMP2	9.87E-23	1.63E-26
CLEC1A	2.92E-13	2.75E-15
HPGD	1.52E-09	4.71E-11
ESAM	5.70E-14	4.31E-16
FMO3	8.21E-09	3.21E-10
PLA1A	1.28E-09	3.89E-11
HLF	5.91E-10	1.60E-11
IFT57	7.35E-11	1.48E-12
KIAA1683	4.80E-08	2.39E-09
PDZD2	1.43E-12	1.72E-14
SORBS1	6.97E-16	2.79E-18
LOC286191	1.42E-16	4.37E-19
PEAR1	1.80E-14	1.16E-16
SLC1A1	1.30E-13	1.11E-15
MS4A8	0.000574	0.000108
SHE	8.61E-10	2.48E-11
CAB39L	3.23E-15	1.70E-17
LOC101927	3.38E-07	2.22E-08
HSPB6	3.12E-09	1.06E-10

AR	1.33E-07	7.69E-09
PDE5A	4.17E-17	1.09E-19
LGI3	1.44E-15	6.65E-18
RASL12	4.54E-11	8.46E-13
LINC00261	7.87E-07	5.77E-08
AQP1	5.61E-10	1.50E-11
SLCO2A1	2.37E-13	2.16E-15
CX3CL1	3.95E-11	7.26E-13
ARHGEF15	6.27E-14	4.82E-16
ANXA3	3.42E-14	2.41E-16
FBLN5	9.63E-16	4.14E-18
CCDC141	3.32E-11	5.96E-13
RPL13AP17	1.78E-09	5.63E-11
CRTAC1	5.27E-07	3.66E-08
GRAMD2	1.41E-06	1.13E-07
AKAP12	1.91E-09	6.09E-11
SULT1C4	1.07E-11	1.62E-13
CLIC3	5.82E-10	1.57E-11
ZNF366	1.14E-11	1.75E-13
IL6	0.00138	0.000296
FAM162B	1.32E-12	1.58E-14
KCNA5	3.58E-11	6.51E-13
FAM184A	5.29E-09	1.93E-10
MAOB	2.42E-10	5.74E-12
CCL15-CCL	8.98E-06	9.26E-07
DAPK2	5.36E-15	3.00E-17
FILIP1	2.24E-12	2.83E-14
THBD	2.02E-11	3.41E-13
ART4	3.59E-13	3.45E-15
BMP5	3.26E-10	8.02E-12
CASKIN2	1.37E-15	6.28E-18
LOC100507	1.57E-10	3.51E-12
PTPN21	3.32E-16	1.17E-18
REEP1	2.46E-10	5.88E-12
ADARB1	1.35E-15	6.13E-18
MBNL1-AS1	1.69E-17	3.55E-20
LAMA3	3.73E-05	4.70E-06
SCN1A	6.55E-06	6.44E-07
TMOD1	8.28E-10	2.37E-11
VGLL3	7.87E-11	1.60E-12
LRRK2	5.65E-07	3.97E-08
PLLP	1.42E-09	4.37E-11
ADGRL3	1.57E-13	1.38E-15

RRAD	1.98E-07	1.21E-08
TNS1	1.33E-15	6.02E-18
ELN	4.69E-06	4.42E-07
CSRNP1	2.63E-11	4.60E-13
PDK4	1.36E-09	4.16E-11
CLEC14A	7.78E-13	8.52E-15
DLC1	3.44E-15	1.84E-17
SHANK3	1.53E-12	1.86E-14
TIE1	2.45E-12	3.14E-14
ANOS1	5.71E-14	4.34E-16
PRG4	6.09E-07	4.32E-08
KCNT2	7.56E-11	1.53E-12
TOX2	5.11E-12	7.22E-14
IL18R1	7.67E-11	1.55E-12
C1QTNF2	1.08E-11	1.65E-13
MYOC	1.92E-11	3.19E-13
ESYT3	4.53E-08	2.24E-09
C9orf135	0.000866	0.000173
SLCO4A1	1.68E-05	1.89E-06
ABLIM3	5.54E-11	1.07E-12
NXF3	1.63E-11	2.63E-13
ABCA9	1.14E-11	1.75E-13
FGD5	1.28E-15	5.75E-18
ADCY4	1.30E-14	8.07E-17
LMO7	6.68E-13	7.14E-15
PIP5K1B	2.93E-12	3.81E-14
GNLY	5.08E-05	6.69E-06
CAPSL	0.000231	3.79E-05
DISP1	1.23E-10	2.64E-12
FGFR2	1.66E-08	7.10E-10
39326	2.32E-14	1.56E-16
WNT2B	4.56E-15	2.50E-17
CFTR	5.28E-05	6.98E-06
SRPX	6.29E-11	1.24E-12
STARD8	4.10E-12	5.60E-14
DPEP2	2.56E-07	1.61E-08
BMP2	2.72E-09	9.07E-11
PENK	9.18E-07	6.93E-08
CNN1	6.01E-08	3.08E-09
LPL	2.41E-11	4.14E-13
WASF3	5.10E-10	1.35E-11
MSRB3	1.32E-11	2.07E-13
AADAC	1.48E-06	1.19E-07

VWA3B	0.000131	1.98E-05
NCALD	4.99E-11	9.39E-13
LIFR	2.56E-11	4.45E-13
SBSPON	8.56E-14	6.87E-16
CAMTA1	2.63E-09	8.72E-11
FEZ1	1.10E-10	2.32E-12
NPR3	7.13E-13	7.70E-15
DAAM2	1.42E-08	5.90E-10
LDLR	1.13E-13	9.46E-16
COX4I2	5.22E-14	3.88E-16
ADHFE1	2.70E-08	1.24E-09
GATA2	1.50E-13	1.31E-15
EMP1	1.19E-10	2.53E-12
MS4A2	2.24E-07	1.39E-08
PLCB4	2.93E-07	1.88E-08
ARHGEF26	4.70E-18	6.61E-21
RAP1A	1.67E-16	5.27E-19
PCDH12	3.87E-13	3.77E-15
FCER1A	0.000109	1.61E-05
C6	0.000226	3.69E-05
ITGA10	5.94E-08	3.04E-09
GUCY1A2	1.76E-13	1.55E-15
ABI3BP	3.01E-12	3.96E-14
GIMAP1	5.29E-13	5.39E-15
FGF2	6.14E-10	1.68E-11
TMEM139	4.52E-13	4.51E-15
SPAG6	0.00218	0.000504
GPX3	1.28E-14	7.87E-17
WWC2	1.01E-15	4.38E-18
SCNN1G	1.79E-07	1.07E-08
SGCD	1.12E-10	2.36E-12
NEDD4L	1.68E-12	2.05E-14
SCNN1B	4.34E-06	4.04E-07
39692	3.55E-16	1.28E-18
ZEB1	5.61E-19	4.31E-22
PRDM11	1.31E-14	8.17E-17
PALMD	1.03E-12	1.18E-14
CXCR2	2.72E-07	1.73E-08
PRELP	1.11E-08	4.48E-10
C1orf116	4.54E-05	5.89E-06
DNAI2	0.000121	1.80E-05
CXorf57	2.36E-14	1.59E-16
PRICKLE2	1.84E-12	2.29E-14

ADGRD1	2.11E-09	6.80E-11
ARGLU1	5.75E-13	5.98E-15
CLEC4M	2.04E-06	1.72E-07
LIMCH1	2.22E-14	1.48E-16
NRN1	1.52E-12	1.84E-14
C7	7.89E-06	7.99E-07
LOC101930	1.18E-13	9.95E-16
ACE	3.31E-09	1.13E-10
THSD4	6.37E-10	1.75E-11
SYNPO2	3.31E-11	5.95E-13
FRMD3	2.85E-15	1.47E-17
DNAJC15	2.92E-11	5.15E-13
PARVB	1.27E-12	1.50E-14
DNALI1	9.96E-09	3.96E-10
SFTPB	1.89E-05	2.17E-06
PREX2	5.84E-12	8.41E-14
RASSF8	4.81E-12	6.73E-14
ACOXL	6.78E-10	1.88E-11
TSLP	1.47E-09	4.53E-11
RGCC	5.99E-14	4.58E-16
NEK5	2.83E-05	3.43E-06
PTPRD	2.72E-07	1.73E-08
PPARGC1A	2.95E-07	1.89E-08
LOC101928	2.20E-05	2.57E-06
NOVA2	5.16E-12	7.33E-14
KLRF1	1.12E-07	6.29E-09
PCOLCE2	2.10E-05	2.44E-06
ERG	1.25E-15	5.53E-18
EP300-AS1	7.47E-10	2.11E-11
BDNF	1.74E-09	5.47E-11
CXCL3	8.32E-06	8.50E-07
MAP1LC3C	3.84E-10	9.73E-12
ATOH8	2.85E-10	6.92E-12
VAPA	1.06E-12	1.22E-14
TMEM204	6.21E-15	3.53E-17
ACACB	5.46E-13	5.60E-15
LINC00472	9.19E-18	1.61E-20
AKAP14	2.00E-04	3.23E-05
ST6GALNAC	1.62E-07	9.59E-09
CXorf36	2.91E-10	7.08E-12
SOCS2	6.64E-09	2.50E-10
EIF5	3.58E-11	6.51E-13
ITPRIP	3.71E-09	1.30E-10

SAMD5	1.82E-08	7.86E-10
IL1A	5.52E-05	7.37E-06
LOC100653	6.01E-07	4.26E-08
CDKN1C	5.18E-12	7.37E-14
S1PR1	1.73E-15	8.27E-18
TGFB2	1.64E-10	3.69E-12
VWF	5.60E-13	5.80E-15
BAALC	4.01E-07	2.70E-08
TTLL7	5.24E-16	1.97E-18
FXYD6	1.71E-11	2.80E-13
CCDC68	1.18E-10	2.51E-12
SEC14L4	1.58E-05	1.76E-06
UST	1.53E-09	4.76E-11
MIR4738	8.69E-14	6.99E-16
GIMAP8	2.20E-14	1.46E-16
FOXF2	7.23E-10	2.04E-11
TMEM74B	6.97E-13	7.49E-15
NDRG2	1.43E-12	1.72E-14
ABHD6	5.33E-13	5.44E-15
GALNT18	6.33E-15	3.61E-17
LINC01140	4.11E-08	2.00E-09
MMRN2	7.03E-14	5.50E-16
ZBBX	0.000557	0.000104
SVEP1	1.33E-14	8.34E-17
CADM1	2.43E-09	7.97E-11
CD93	5.33E-14	4.00E-16
TPSB2	0.000213	3.46E-05
GIPC2	1.66E-07	9.87E-09
TMEM35A	1.93E-08	8.43E-10
ADAMTS9	0.00012	1.79E-05
NOTCH4	7.03E-14	5.51E-16
SLC26A9	0.000224	3.66E-05
LAMP3	6.13E-09	2.28E-10
ARRB1	6.17E-13	6.48E-15
PTX3	0.000388	6.88E-05
CASZ1	1.26E-10	2.72E-12
LRRC46	6.40E-05	8.76E-06
BEX1	5.47E-09	2.00E-10
ZNF423	1.72E-10	3.88E-12
RERG	1.21E-09	3.65E-11
NR4A3	0.00136	0.000293
CLDN11	1.52E-09	4.69E-11
FAXDC2	3.87E-08	1.87E-09

SCEL	4.06E-07	2.73E-08
AGBL1	3.20E-07	2.07E-08
MYCT1	8.05E-14	6.44E-16
ECSCR	9.42E-16	4.01E-18
C10orf10	9.71E-09	3.85E-10
MAL	2.41E-06	2.08E-07
TMTC1	1.69E-09	5.29E-11
S100A10	1.64E-07	9.73E-09
CCL23	2.02E-08	8.86E-10
EYA4	6.27E-08	3.23E-09
HIF3A	1.41E-07	8.18E-09
SOX6	1.09E-07	6.15E-09
CFD	1.91E-09	6.09E-11
SSBP2	7.88E-12	1.16E-13
PTN	5.05E-09	1.83E-10
CFAP52	9.70E-05	1.40E-05
PPP1R15A	3.56E-12	4.77E-14
COX7A1	7.48E-13	8.15E-15
CFAP43	5.76E-05	7.73E-06
POU6F1	4.87E-13	4.92E-15
BMP6	4.04E-08	1.96E-09
LOC102724	2.32E-10	5.49E-12
DYNC1I2	5.35E-10	1.42E-11
RUNX1T1	3.27E-07	2.13E-08
LINC01268	9.98E-11	2.08E-12
TSPAN3	1.12E-09	3.35E-11
CXCL2	7.50E-08	3.97E-09
MGAM	1.34E-07	7.76E-09
GSTM3	3.21E-10	7.91E-12
PLEKHH2	3.37E-13	3.21E-15
KLF9	9.38E-09	3.71E-10
NPNT	1.14E-12	1.33E-14
APOH	8.40E-05	1.19E-05
CATSPERD	0.000216	3.52E-05
38231	3.22E-12	4.26E-14
COBL	3.84E-08	1.85E-09
DMBT1	0.00347	0.000864
TCTEX1D1	8.78E-10	2.53E-11
HK3	3.00E-04	5.12E-05
DUOXA1	3.37E-07	2.21E-08
WFS1	1.98E-16	6.56E-19
ADAMTS1	1.44E-07	8.40E-09
SFRP1	0.000198	3.19E-05

IL1B	0.000329	5.70E-05
QKI	1.27E-12	1.50E-14
PALM2-AKA	4.70E-18	6.59E-21
FRY	9.54E-13	1.08E-14
PTGS2	0.000587	0.000111
MICALCL	1.59E-09	4.96E-11
SHANK2	1.98E-07	1.21E-08
NES	2.57E-11	4.48E-13
NAPSA	0.00359	0.000901
ABCA6	4.78E-10	1.26E-11
PID1	3.37E-08	1.59E-09
PROK2	3.30E-05	4.08E-06
LOC729966	1.13E-08	4.57E-10
C11orf88	0.000339	5.90E-05
SELP	9.71E-09	3.85E-10
CNTNAP3B	2.76E-10	6.68E-12
TCEAL7	9.08E-09	3.58E-10
TEKT1	0.000465	8.48E-05
C16orf89	0.00127	0.000269
IL18RAP	3.13E-06	2.78E-07
HBEGF	2.65E-09	8.80E-11
MEIS1	3.08E-09	1.04E-10
PLCE1	4.05E-10	1.04E-11
SHC3	6.63E-08	3.44E-09
HMGCLL1	1.85E-10	4.21E-12
ACTG2	9.83E-07	7.50E-08
DPT	4.29E-07	2.90E-08
IL17D	1.25E-11	1.95E-13
VSTM4	3.45E-12	4.60E-14
C8orf34-A	3.45E-05	4.30E-06
CELF2	6.02E-12	8.69E-14
CLU	8.37E-07	6.22E-08
C10orf54	9.00E-15	5.29E-17
STC2	9.55E-05	1.38E-05
CYP2B7P	0.000142	2.16E-05
MMRN1	1.37E-05	1.50E-06
CCL2	8.94E-06	9.21E-07
MTURN	1.40E-08	5.81E-10
FGF18	3.02E-10	7.37E-12
SLC16A5	8.58E-12	1.27E-13
ARNT2	1.29E-05	1.40E-06
NDNF	2.89E-07	1.85E-08
MOCS1	1.95E-09	6.22E-11

C1orf115	1.12E-09	3.35E-11
SLC24A3	7.91E-07	5.81E-08
TPSAB1	2.89E-05	3.50E-06
RBPMS	1.76E-07	1.05E-08
ST6GALNAC	3.88E-09	1.36E-10
FAM216B	0.000302	5.16E-05
TYRP1	1.02E-05	1.07E-06
MACROD2	0.00155	0.000338
CEBPD	2.53E-10	6.07E-12
MAFF	1.70E-10	3.84E-12
CDHR3	2.96E-05	3.60E-06
LOC101928	0.00175	0.000389
IL20RA	8.25E-05	1.17E-05
TMEM237	9.20E-11	1.90E-12
LGALSL	4.48E-13	4.45E-15
NHSL2	8.46E-08	4.57E-09
GPRIN2	2.56E-09	8.45E-11
FPR2	7.28E-05	1.01E-05
DENND3	1.20E-16	3.59E-19
GABRB2	1.04E-05	1.10E-06
CYYR1	2.43E-12	3.10E-14
CBFA2T3	2.17E-11	3.69E-13
OLFML2A	1.10E-07	6.16E-09
FAM65A	7.71E-12	1.13E-13
UACA	1.73E-11	2.84E-13
JPH2	7.20E-09	2.76E-10
DOK6	1.15E-17	2.19E-20
FOXP1	4.35E-13	4.31E-15
FSTL1	1.15E-07	6.49E-09
ARAP3	2.29E-14	1.53E-16
SCARA3	1.78E-08	7.64E-10
CMTM2	1.63E-06	1.33E-07
TPSB2T	5.53E-05	7.38E-06
ICAM1	8.00E-08	4.28E-09
EPAS1	4.16E-18	5.25E-21
LONRF1	1.03E-11	1.56E-13
RIMS3	4.17E-07	2.81E-08
AFAP1L1	2.27E-10	5.36E-12
RGS13	3.31E-06	2.97E-07
PGM5-AS1	1.53E-14	9.74E-17
COL6A5	6.74E-06	6.67E-07
CDH6	3.27E-06	2.92E-07
PALD1	3.89E-09	1.37E-10

LSAMP	1.17E-10	2.48E-12
SMAD9	1.51E-09	4.69E-11
AMOTL1	1.41E-15	6.50E-18
LOC101928	1.13E-13	9.48E-16
SASH1	3.15E-17	7.67E-20
PRSS12	5.34E-05	7.09E-06
ARHGAP31	1.82E-15	8.81E-18
IL33	4.16E-08	2.03E-09
PRAM1	1.75E-07	1.05E-08
PTGIR	1.80E-11	2.97E-13
COL13A1	2.22E-09	7.20E-11
CLEC12A	2.59E-06	2.25E-07
ARHGAP20	4.86E-06	4.59E-07
GADD45B	1.64E-07	9.74E-09
HDC	2.46E-07	1.53E-08
SFTA3	0.00166	0.000366
TIMP3	2.36E-14	1.59E-16
DCDC2	9.78E-06	1.02E-06
HEYL	6.39E-10	1.76E-11
LINC00551	2.82E-05	3.42E-06
GFOD1	8.87E-10	2.56E-11
S1PR5	1.02E-08	4.04E-10
MMP19	2.66E-05	3.20E-06
KCNAB1	1.20E-08	4.88E-10
TUB	2.91E-07	1.86E-08
RSPH1	0.00474	0.00124
CYP2B7P	3.27E-05	4.04E-06
ADAMTS15	1.47E-06	1.18E-07
BCL6B	1.31E-12	1.55E-14
P2RY1	1.89E-10	4.33E-12
PRDM16	5.02E-07	3.47E-08
KIAA1324L	1.40E-07	8.12E-09
SPIDR	3.34E-10	8.28E-12
PLXNA2	1.32E-09	4.02E-11
HOXA5	2.72E-06	2.38E-07
SCN2B	5.74E-10	1.54E-11
PDLIM2	2.03E-15	1.01E-17
NTRK3	4.63E-11	8.66E-13
ULK2	7.23E-11	1.45E-12
DKK3	1.51E-09	4.68E-11
LEFTY1	4.53E-08	2.24E-09
TMEM47	2.88E-13	2.70E-15
C4BPA	0.00445	0.00116

MIR22	1.43E-09	4.38E-11
C1orf21	4.86E-10	1.28E-11
FOXA2	0.000191	3.05E-05
RND1	0.00177	0.000395
SPRY4-IT1	5.29E-09	1.93E-10
ALPL	6.34E-05	8.65E-06
OLFM1	2.31E-07	1.43E-08
DNM3	1.33E-07	7.69E-09
PTRF	7.81E-15	4.53E-17
DNASE2B	0.000384	6.81E-05
PADI4	5.75E-06	5.55E-07
PKIA	3.43E-09	1.18E-10
EFCAB1	0.000478	8.76E-05
NEDD9	6.28E-13	6.63E-15
KLHL41	1.51E-08	6.36E-10
CAMK2N1	2.96E-10	7.22E-12
C11orf21	2.40E-08	1.08E-09
LRCH2	9.85E-09	3.91E-10
NFATC1	2.75E-07	1.75E-08
PPARG	5.14E-06	4.89E-07
UBASH3B	5.48E-06	5.25E-07
PHACTR1	2.04E-11	3.43E-13
ROR1	9.21E-10	2.68E-11
FBLN1	7.32E-08	3.85E-09
FSTL3	5.46E-07	3.81E-08
ACSS3	3.44E-07	2.26E-08
DNAI1	0.00127	0.000269
LOC101929	1.87E-06	1.56E-07
RADIL	7.04E-14	5.53E-16
SPRYD7	3.52E-12	4.71E-14
HEG1	7.13E-13	7.68E-15
TRPC6	8.07E-05	1.14E-05
FAM13C	4.07E-09	1.44E-10
LILRA2	4.05E-06	3.72E-07
TRPV2	8.22E-08	4.42E-09
GRASP	1.53E-12	1.86E-14
SPATA18	0.000398	7.08E-05
COPG2IT1	1.99E-10	4.60E-12
COL21A1	9.68E-07	7.35E-08
MFSD2A	1.44E-06	1.15E-07
EIF3M	2.24E-09	7.29E-11
NECTIN3	1.05E-08	4.22E-10
TACC1	1.75E-14	1.13E-16

ADGRG6	4.79E-11	8.98E-13
FGF14	1.90E-08	8.25E-10
DCC	3.88E-08	1.87E-09
GSTM5	6.08E-10	1.66E-11
KCNMB4	3.83E-05	4.85E-06
WDR78	2.74E-05	3.31E-06
MIR29C	1.89E-06	1.57E-07
RPL23AP32	7.65E-13	8.36E-15
PRDM6	5.54E-07	3.88E-08
LINC00622	9.72E-06	1.01E-06
CPED1	7.91E-14	6.29E-16
FZD4	5.24E-13	5.32E-15
EFHB	1.55E-05	1.72E-06
CACNA1C	6.26E-07	4.45E-08
PDGFB	3.78E-10	9.54E-12
SPEF2	4.99E-05	6.56E-06
DST	5.36E-15	3.00E-17
WWC1	7.65E-07	5.59E-08
FAT4	4.07E-09	1.44E-10
ARHGEF10	1.98E-15	9.80E-18
MAATS1	1.32E-06	1.04E-07
ITIH3	2.56E-09	8.42E-11
ICAM2	2.50E-13	2.31E-15
RBMS2	2.04E-14	1.33E-16
BRE-AS1	0.000342	5.95E-05
CD34	6.98E-08	3.64E-09
GATA6	1.52E-08	6.41E-10
LRRC49	2.17E-10	5.08E-12
CSPG4	3.07E-10	7.52E-12
PRICKLE1	6.29E-07	4.47E-08
KAZN	4.38E-11	8.12E-13
FFAR2	0.00724	0.00205
NEXN	7.69E-09	2.98E-10
CLDND1	1.51E-09	4.69E-11
ATF3	1.21E-05	1.30E-06
RAB11A	3.41E-16	1.21E-18
CA3	9.27E-08	5.08E-09
PEG10	0.000245	4.07E-05
ZFPM2	8.68E-08	4.70E-09
PHACTR3	0.00328	0.000811
PZP	6.41E-09	2.40E-10
FLRT3	9.41E-06	9.78E-07
TSPAN12	4.13E-12	5.65E-14

PCDH17	5.76E-09	2.13E-10
CAPN3	3.65E-06	3.30E-07
F8	6.71E-09	2.54E-10
ITM2A	1.60E-10	3.57E-12
ARHGEF37	1.62E-06	1.32E-07
SHROOM2	1.05E-07	5.82E-09
BMPR2	8.71E-18	1.50E-20
SLC18A2	0.000135	2.04E-05
CNR1	3.03E-05	3.70E-06
KLHL29	4.53E-08	2.24E-09
CPAMD8	1.27E-07	7.25E-09
OLFML1	6.83E-09	2.59E-10
GNL1	1.95E-11	3.25E-13
PRKCQ	2.50E-06	2.16E-07
ZDHHC11B	6.13E-06	5.98E-07
SGCE	1.80E-10	4.09E-12
MYL9	1.62E-09	5.06E-11
CFAP70	0.000472	8.63E-05
ATP10A	3.57E-07	2.36E-08
TEX14	6.73E-07	4.83E-08
TMEM246	1.71E-07	1.02E-08
TBX5	3.34E-09	1.15E-10
CTNNAL1	2.77E-17	6.54E-20
SEMA3E	1.36E-06	1.08E-07
MYADM	3.22E-12	4.26E-14
GIMAP6	1.45E-11	2.30E-13
LRRN4CL	6.46E-06	6.34E-07
CAT	2.07E-16	6.93E-19
IRX1	2.56E-06	2.22E-07
SAMD4A	1.24E-08	5.06E-10
LOC100506	7.42E-08	3.92E-09
PPARGC1B	1.51E-07	8.90E-09
LOC101928	1.62E-05	1.82E-06
CLEC4E	0.00126	0.000266
KIAA0040	5.83E-15	3.28E-17
LOC100506	6.42E-09	2.41E-10
AMIGO1	1.40E-08	5.83E-10
PEG3	1.72E-06	1.41E-07
FGR	6.99E-08	3.65E-09
CBX7	5.03E-11	9.48E-13
MAP2	1.16E-05	1.24E-06
OTUD1	1.13E-14	6.76E-17
SNORD54	2.02E-11	3.40E-13

SYNPO	5.85E-11	1.14E-12
NME5	0.000123	1.84E-05
TMEM39A	1.37E-11	2.15E-13
LMO2	3.85E-14	2.75E-16
LOC285812	1.61E-09	5.00E-11
PTGDS	8.58E-06	8.80E-07
CCDC50	3.76E-12	5.06E-14
SLC39A8	2.68E-10	6.48E-12
ADGRF5	1.94E-07	1.18E-08
PKIG	4.32E-13	4.27E-15
PIEZO2	2.41E-05	2.86E-06
LRP2	0.00146	0.000315
WDFY3-AS2	6.61E-07	4.73E-08
LINC00961	9.85E-10	2.89E-11
AIF1L	1.50E-05	1.66E-06
MAOA	1.47E-07	8.62E-09
LHFP	1.77E-12	2.19E-14
PRTG	6.88E-05	9.50E-06
SOCS3	1.29E-06	1.02E-07
ARHGAP44	5.99E-09	2.22E-10
CDKL2	0.00073	0.000142
RECK	3.16E-11	5.61E-13
CPA3	3.70E-05	4.65E-06
SCD5	3.88E-09	1.36E-10
COL14A1	0.00226	0.000525
LOC105379	5.42E-11	1.04E-12
FILIP1L	7.27E-10	2.05E-11
LARP6	7.19E-10	2.02E-11
MYH10	4.21E-13	4.14E-15
GIMAP1-GI	3.18E-10	7.83E-12
PTPRN2	3.95E-06	3.63E-07
OSR1	3.68E-07	2.44E-08
NKD1	4.52E-09	1.62E-10
MYOM2	9.13E-08	4.99E-09
NKD2	2.43E-08	1.09E-09
SOX5	4.54E-11	8.44E-13
SPNS2	4.42E-09	1.58E-10
NRGN	0.000728	0.000142
RAB11FIP1	3.17E-15	1.66E-17
C1orf194	0.000606	0.000115
P2RY14	2.47E-08	1.12E-09
SIRPB1	5.76E-06	5.56E-07
CD1E	0.0011	0.000228

SH2D1B	6.54E-06	6.44E-07
SPG20	1.32E-10	2.85E-12
RGS9	1.05E-08	4.19E-10
COBLL1	2.55E-08	1.16E-09
MORN5	0.00151	0.000328
FREM2	0.000121	1.81E-05
SRSF3	3.25E-12	4.31E-14
NT5DC1	1.57E-11	2.52E-13
MRGPRF	1.50E-05	1.65E-06
BDH2	1.09E-09	3.23E-11
TNS2	2.29E-11	3.92E-13
PECAM1	3.70E-13	3.58E-15
FAR2	4.51E-06	4.22E-07
CDON	1.75E-05	1.98E-06
ABCG2	4.27E-08	2.09E-09
PDE2A	1.81E-08	7.82E-10
PLCXD3	7.78E-05	1.09E-05
SLC6A16	1.28E-06	1.01E-07
ETV1	4.88E-09	1.75E-10
LOC101927	7.29E-05	1.01E-05
RPL15	7.19E-10	2.01E-11
MCC	5.55E-09	2.04E-10
NR2F2	3.37E-07	2.20E-08
NFIA	4.38E-11	8.13E-13
LOC101927	4.83E-10	1.27E-11
PTPRG	1.37E-14	8.62E-17
PODXL	8.94E-16	3.74E-18
REPS2	1.91E-06	1.60E-07
ECM2	1.39E-07	8.08E-09
LOC643733	1.58E-10	3.52E-12
MDH1B	0.0013	0.000277
SOBP	3.03E-07	1.95E-08
COL4A6	0.00049	9.01E-05
CA5B	5.85E-11	1.14E-12
BMPR1B	0.0026	0.000618
LOC102724	1.83E-05	2.08E-06
RNF144B	3.41E-13	3.26E-15
ROPN1L	0.00083	0.000165
PDLIM3	8.60E-07	6.42E-08
RASGRP2	8.06E-09	3.14E-10
LMCD1	8.58E-09	3.36E-10
USP44	3.04E-06	2.69E-07
KANK2	9.97E-13	1.14E-14

H2AFJ	3.51E-08	1.67E-09
AK1	9.48E-08	5.20E-09
ARHGEF12	2.35E-13	2.14E-15
CNTNAP3B	1.50E-06	1.21E-07
RBP4	0.0021	0.000482
SLC25A25	2.29E-10	5.42E-12
SPATS2L	1.59E-08	6.76E-10
PAQR5	3.95E-05	5.03E-06
SFTA2	0.00994	0.00298
HLX	1.53E-09	4.76E-11
CD101	9.13E-07	6.88E-08
ADAMTSL4	0.000295	5.03E-05
C3orf70	2.96E-05	3.60E-06
CCRL2	8.75E-06	9.00E-07
EHD2	8.85E-11	1.81E-12
OSCAR	6.85E-05	9.45E-06
PGAP1	2.49E-08	1.13E-09
HAS1	0.00872	0.00255
THRB	6.89E-09	2.62E-10
WAC	1.81E-11	2.98E-13
LOC101927	1.37E-08	5.69E-10
SYT15	2.14E-10	4.99E-12
SLC2A12	2.10E-07	1.29E-08
CA2	1.37E-06	1.10E-07
TPM3	1.48E-09	4.55E-11
ANK2	1.13E-06	8.73E-08
SELENBP1	1.05E-06	8.05E-08
ETS2	1.99E-09	6.38E-11
HOXA4	1.05E-09	3.11E-11
FYN	2.39E-07	1.49E-08
CFAP157	7.00E-04	0.000135
INMT-FAM1	7.91E-07	5.81E-08
EVA1A	1.91E-05	2.19E-06
SNX25	4.94E-10	1.30E-11
RAPGEF4	1.86E-12	2.32E-14
FCN1	1.87E-07	1.13E-08
MCOLN3	6.44E-06	6.31E-07
HSPB2	1.94E-08	8.46E-10
COL4A3	1.41E-07	8.25E-09
PTCHD1	1.14E-05	1.22E-06
LINC00702	3.13E-08	1.46E-09
NLRP3	0.00111	0.00023
DLEC1	0.00362	0.00091

PARD3B	1.33E-13	1.15E-15
VWA3A	1.44E-06	1.15E-07
SDC2	1.76E-09	5.56E-11
RHOBTB2	6.11E-06	5.95E-07
CFL2	6.90E-15	3.99E-17
CACHD1	0.000166	2.60E-05
PRKG1	4.57E-10	1.19E-11
OASL	3.18E-05	3.92E-06
BMX	5.80E-09	2.15E-10
CD274	1.52E-05	1.69E-06
RAPGEF5	1.32E-13	1.14E-15
ADTRP	0.000368	6.47E-05
DOCK4	4.87E-13	4.91E-15
FGF14-AS2	4.82E-09	1.73E-10
UNC5C	1.33E-06	1.06E-07
APOBEC3A_	0.000183	2.90E-05
LOC102725	2.16E-08	9.54E-10
LOC401317	6.02E-06	5.84E-07
GGTLC1	2.99E-05	3.64E-06
CNTN4	7.79E-08	4.15E-09
ROS1	0.00281	0.000678
SETBP1	3.26E-07	2.12E-08
KLF2	5.49E-09	2.01E-10
PARVA	8.25E-14	6.61E-16
AHNAK	1.19E-14	7.14E-17
KIAA1211L	4.06E-08	1.97E-09
RAVER2	2.37E-08	1.07E-09
EPB41L5	2.25E-09	7.34E-11
BEX5	2.45E-05	2.91E-06
CYP4Z1	0.000249	4.14E-05
JAM3	3.64E-10	9.18E-12
ENG	4.01E-10	1.02E-11
SMCO3	1.89E-05	2.16E-06
CST6	0.00397	0.00101
HCAR3	0.00941	0.00279
SH3D19	8.98E-16	3.78E-18
CSF3R	0.000561	0.000105
TMEM110	1.10E-11	1.68E-13
CTSW	0.00189	0.000428
TEF	1.32E-05	1.44E-06
TTC28	4.38E-14	3.21E-16
HYAL2	7.09E-11	1.42E-12
VSNL1	2.02E-06	1.70E-07

MACF1	1.29E-15	5.79E-18
KCNJ16	0.00506	0.00134
ZNF106	4.29E-18	5.65E-21
CUL3	3.59E-13	3.46E-15
CLSTN2	0.000116	1.73E-05
ATP13A4	0.000595	0.000113
CYBRD1	2.40E-10	5.70E-12
KIAA0368	4.35E-11	8.06E-13
KDR	3.33E-08	1.57E-09
FBP1	1.73E-05	1.95E-06
FADS3	1.95E-08	8.54E-10
BEND7	4.89E-07	3.36E-08
AOX1	1.77E-07	1.06E-08
SLC27A3	2.43E-08	1.10E-09
PLPP3	1.02E-11	1.55E-13
KIT	0.000139	2.12E-05
KCNN2	6.69E-05	9.20E-06
TMOD3	1.88E-08	8.16E-10
GATA6-AS1	2.28E-08	1.02E-09
LOC101930	3.70E-08	1.77E-09
ST8SIA1	1.03E-08	4.11E-10
CDKN2D	1.95E-10	4.48E-12
PLN	0.000295	5.03E-05
OBFC1	5.82E-10	1.57E-11
SPARCL1	4.05E-10	1.04E-11
LOC101927	1.42E-07	8.27E-09
HDAC4	4.04E-08	1.96E-09
STAC	2.68E-05	3.23E-06
PPP1R12B	1.80E-10	4.11E-12
MEOX2	0.00022	3.59E-05
ZNF608	5.69E-07	4.00E-08
DIRAS3	0.000319	5.50E-05
PPP1R3C	0.00219	0.000508
PKHD1L1	0.000346	6.02E-05
ADGRE5	1.27E-10	2.73E-12
USP47	2.26E-11	3.87E-13
ADGRG2	0.000569	0.000107
HSPB7	9.23E-08	5.05E-09
IL7R	1.67E-07	9.90E-09
CNRIP1	1.31E-08	5.37E-10
TRIOBP	4.73E-15	2.60E-17
MYO15B	1.26E-07	7.24E-09
LOC101930	9.39E-10	2.74E-11

KCNE1	1.53E-05	1.71E-06
B3GALNT1	1.22E-09	3.69E-11
JUN	6.53E-09	2.45E-10
SNX2	1.63E-10	3.65E-12
PRDM5	2.14E-09	6.93E-11
IL3RA	5.54E-08	2.81E-09
EPB41L2	1.85E-08	8.02E-10
PCM1	5.63E-11	1.09E-12
ACAA2	9.18E-08	5.02E-09
B3GALT2	1.62E-09	5.07E-11
RWDD1	1.44E-11	2.28E-13
ARMCX4	3.44E-08	1.63E-09
VSIG4	6.36E-05	8.68E-06
ADGRE1	3.35E-05	4.16E-06
PIK3C3	9.17E-10	2.66E-11
CLEC2B	9.07E-05	1.30E-05
ALDH1A1	7.83E-08	4.17E-09
FHDC1	1.25E-09	3.79E-11
NR3C2	1.70E-07	1.01E-08
BMPER	4.52E-12	6.29E-14
NPC1	4.56E-08	2.26E-09
MYLK	3.71E-14	2.63E-16
IDH3A	3.17E-09	1.08E-10
APOBR	1.58E-06	1.28E-07
INPP5A	1.36E-16	4.12E-19
FAM134B	3.69E-06	3.35E-07
DCUN1D5	4.88E-12	6.84E-14
CD83	9.02E-07	6.80E-08
MID2	5.84E-11	1.14E-12
CCDC78	0.00426	0.0011
PCDHGA1	5.70E-08	2.91E-09
RPS6KA2	7.14E-10	2.00E-11
ZNF662	1.31E-05	1.43E-06
ATN1	0.00472	0.00124
TNFRSF10D	1.66E-06	1.35E-07
LCAT	4.13E-09	1.46E-10
KDM6B	9.71E-07	7.39E-08
AKT3	8.42E-13	9.35E-15
RNF180	5.56E-08	2.82E-09
PLA2G4C	3.51E-07	2.31E-08
LTK	5.24E-05	6.93E-06
ZEB2	1.05E-08	4.21E-10
OSBPL6	8.10E-07	5.98E-08

DAW1	0.00114	0.000238
MXRA7	1.00E-07	5.57E-09
ERP44	4.47E-11	8.32E-13
RRAS	4.85E-12	6.79E-14
PLSCR4	5.72E-11	1.11E-12
HACD1	1.76E-06	1.45E-07
SPATA6	2.49E-07	1.56E-08
AATK	1.13E-09	3.35E-11
RNF128	0.002	0.000457
DRC3	6.95E-05	9.61E-06
TMEM231	0.000127	1.91E-05
CLCN4	2.46E-10	5.87E-12
PREX1	6.95E-09	2.64E-10
PHLDB2	1.38E-07	8.04E-09
FLI1	5.42E-09	1.98E-10
SLCO4C1	0.000254	4.24E-05
CARMNM	5.76E-05	7.73E-06
RTN1	7.29E-08	3.84E-09
HLA-E	1.50E-12	1.81E-14
SCTR	0.0035	0.000874
SNX1	1.35E-12	1.61E-14
HGF	1.14E-07	6.46E-09
MAGI2-AS3	1.20E-08	4.89E-10
CXCL1	0.00673	0.00188
RSPH9	0.000769	0.000151
SPRY4	8.21E-10	2.34E-11
PPP4R4	5.02E-07	3.47E-08
PIFO	0.00242	0.000569
FKBP1B	8.49E-08	4.58E-09
IFRD1	3.34E-06	3.00E-07
LTBP2	1.39E-07	8.06E-09
PTPRM	1.63E-09	5.10E-11
MMP23A	0.00185	0.000417
NRG1	2.32E-06	1.99E-07
CRIP1	5.19E-07	3.60E-08
TBC1D1	1.79E-09	5.66E-11
BAIAP2	6.75E-08	3.51E-09
ARHGEF40	2.44E-10	5.81E-12
ATP8A2	0.000189	3.02E-05
NHSL1	3.78E-09	1.32E-10
ARHGAP28	9.24E-05	1.33E-05
SGIP1	1.37E-08	5.70E-10
ARC	2.12E-06	1.80E-07

IGFBP6	0.00013	1.96E-05
FAM49A	5.73E-07	4.03E-08
LAMA2	1.17E-06	9.08E-08
HAS2	0.0045	0.00117
LOC101929	7.89E-13	8.66E-15
PDZRN3	1.49E-07	8.72E-09
APC	7.59E-11	1.53E-12
MFAP3L	0.000307	5.27E-05
LOC285147	2.10E-09	6.78E-11
SOX18	0.000776	0.000153
LOC101930	0.000292	4.97E-05
KLF6	2.43E-12	3.09E-14
PWAR6	1.84E-05	2.10E-06
NLRC4	1.98E-05	2.28E-06
NAALADL1	6.39E-06	6.26E-07
GAS6	2.29E-07	1.43E-08
AGBL2	0.0044	0.00114
PEAK1	1.22E-12	1.43E-14
CD1C	0.000315	5.42E-05
RAPGEF3	3.38E-08	1.60E-09
AGR3	0.00398	0.00101
NOV	3.03E-07	1.95E-08
EPHX2	2.09E-05	2.43E-06
RMST	0.00148	0.000321
SCAI	1.17E-10	2.50E-12
TGFBR2	7.94E-14	6.33E-16
SIGLEC17P	8.62E-07	6.44E-08
MEIS2	4.34E-09	1.55E-10
UBE2I	4.20E-08	2.05E-09
CDKL1	3.50E-10	8.75E-12
BVES	3.59E-08	1.71E-09
HEY2	1.59E-07	9.38E-09
CCDC17	0.00095	0.000192
CNTN1	1.94E-05	2.22E-06
LY75-CD30	7.12E-12	1.04E-13
NLRP1	6.50E-06	6.38E-07
WNT9A	2.68E-06	2.34E-07
DNAJC6	6.92E-06	6.86E-07
MAP4	4.08E-12	5.57E-14
OMD	0.000483	8.86E-05
AGAP11	2.17E-05	2.53E-06
NAALAD2	2.70E-09	9.00E-11
LOC101928	3.62E-08	1.73E-09

ZDHHC2	8.39E-07	6.24E-08
LOC100506	2.66E-05	3.19E-06
CRIP2	3.13E-07	2.03E-08
FAM43A	4.76E-06	4.49E-07
MT1E	5.82E-07	4.10E-08
DPYSL2	3.06E-11	5.42E-13
CDKN2B	3.65E-05	4.59E-06
NTN4	4.72E-07	3.22E-08
HSPH1	1.17E-06	9.12E-08
PPP2CA	1.03E-13	8.46E-16
APCDD1	5.12E-06	4.87E-07
ARHGAP29	5.82E-11	1.13E-12
AMOTL2	2.09E-10	4.85E-12
COL4A5	0.00101	0.000208
HEY1	0.000625	0.000119
C8orf60	3.92E-07	2.63E-08
CD52	3.91E-05	4.96E-06
ADAM33	2.25E-08	1.00E-09
DCSTAMP	0.00962	0.00287
LOC729680	9.15E-06	9.46E-07
LOC202025	5.87E-07	4.14E-08
GPR4	8.71E-08	4.73E-09
SAXO2	0.00159	0.00035
SHROOM3	2.27E-07	1.41E-08
QSOX1	0.000109	1.60E-05
GLIPR2	5.84E-09	2.16E-10
STEAP4	0.00128	0.000271
NDUFAF4	1.33E-07	7.69E-09
LHFPL3-AS	2.58E-07	1.62E-08
STARD4	1.75E-08	7.48E-10
RAB14	2.84E-08	1.31E-09
CNTN6	2.65E-10	6.39E-12
PRF1	0.000325	5.62E-05
ZMYND10	0.00239	0.000559
PHACTR2	3.81E-14	2.71E-16
LOC100507	0.000126	1.90E-05
SPTBN1	9.77E-20	5.90E-23
VANGL2	0.00499	0.00132
JAML	1.31E-05	1.42E-06
GPD1L	3.72E-09	1.30E-10
LOC100288	1.59E-08	6.72E-10
RCAN1	1.41E-07	8.18E-09
SOX13	3.39E-06	3.05E-07

PPM1L	2.17E-06	1.85E-07
CTIF	2.43E-09	7.97E-11
MSX1	5.70E-06	5.49E-07
PLCL1	2.06E-08	9.05E-10
LRRC4	7.66E-07	5.60E-08
MRC1	8.01E-06	8.13E-07
DTNA	0.000196	3.15E-05
LEPR	5.46E-07	3.81E-08
DLL1	0.000118	1.76E-05
GBP4	7.17E-06	7.15E-07
C8orf88	3.03E-08	1.41E-09
TEKT2	0.000382	6.75E-05
AHRR	0.00159	0.00035
PPP1R14C	0.000824	0.000164
PLA2G5	0.000162	2.52E-05
CFLAR	3.38E-13	3.22E-15
37316	3.57E-12	4.79E-14
CFP	7.81E-11	1.58E-12
CAPS2	7.52E-06	7.56E-07
EBF1	1.54E-07	9.08E-09
HIVEP3	1.94E-05	2.23E-06
RBPMS2	0.00113	0.000236
LRRC57	1.44E-15	6.68E-18
FBXO15	0.000204	3.29E-05
MN1	0.000117	1.75E-05
ATP5S	3.19E-12	4.22E-14
FOS	1.28E-05	1.38E-06
SNRK	5.32E-15	2.96E-17
USHBP1	7.55E-07	5.50E-08
MLF1	1.99E-05	2.29E-06
SAP18	4.85E-14	3.59E-16
PHYHD1	5.41E-06	5.18E-07
OLR1	0.00112	0.000233
ST6GALNAC	2.23E-09	7.26E-11
CYR61	4.35E-05	5.61E-06
SLC44A1	1.51E-14	9.55E-17
DMD	5.16E-08	2.59E-09
CSF1	1.07E-07	5.98E-09
CEP126	4.37E-07	2.97E-08
NCAM1	0.000535	9.97E-05
PNPLA6	9.17E-07	6.92E-08
F10	3.34E-12	4.45E-14
C4orf22	0.00112	0.000232

ODAM	3.85E-05	4.87E-06
EGFEM1P	7.18E-06	7.17E-07
GJA5	0.000316	5.45E-05
CFAP221	0.00317	0.000779
DAB2IP	5.10E-12	7.20E-14
NFIX	3.60E-06	3.26E-07
LOC101927	0.00404	0.00103
FAM65B	3.50E-05	4.37E-06
DYNC2H1	3.39E-08	1.60E-09
ZHX3	8.45E-10	2.42E-11
LRRC2	1.53E-08	6.44E-10
MAPRE2	1.75E-10	3.97E-12
ARID5A	6.93E-07	5.00E-08
COL4A4	0.000253	4.21E-05
SH3BP5	2.58E-12	3.33E-14
ALOX15B	0.0098	0.00293
ADCY9	7.75E-08	4.12E-09
NR2F1	3.14E-07	2.04E-08
FLT4	3.28E-05	4.06E-06
CABLES1	3.33E-06	2.99E-07
C11orf96	1.15E-05	1.22E-06
PPID	1.73E-07	1.03E-08
PCDHB4	3.01E-06	2.66E-07
TCF7L1	1.24E-05	1.33E-06
C10orf25	5.25E-09	1.91E-10
KCTD15	0.000201	3.24E-05
CASC1	0.00188	0.000423
LOC100506	5.50E-08	2.78E-09
ARHGAP24	5.61E-10	1.50E-11
PBX1	6.09E-09	2.27E-10
NAP1L5	3.93E-07	2.64E-08
HHIP-AS1	0.00139	3.00E-04
PITPNM2	9.39E-06	9.75E-07
LTC4S	1.49E-08	6.27E-10
GIMAP7	3.13E-07	2.03E-08
TNIP1	1.92E-08	8.37E-10
DENND2C	1.04E-07	5.81E-09
ANKRD20A1	3.51E-07	2.31E-08
LOC728392	1.77E-06	1.46E-07
PLAC8	3.52E-06	3.18E-07
ALOX5	2.20E-06	1.87E-07
LOC101930	0.00594	0.00162
ZFP36	6.00E-07	4.24E-08

ELAVL1	7.83E-08	4.17E-09
IL11RA	4.09E-10	1.05E-11
FERMT2	4.74E-13	4.76E-15
ARL6	5.86E-11	1.15E-12
PACRG	0.00127	0.000268
CYGB	0.000717	0.000139
TBXA2R	3.20E-07	2.08E-08
RBP2	1.66E-05	1.87E-06
ZDHHC14	3.30E-11	5.92E-13
ID1	1.81E-05	2.06E-06
KCNRG	0.000179	2.83E-05
PTGER4	4.46E-07	3.03E-08
SIK2	4.89E-13	4.94E-15
SYN2	4.21E-06	3.89E-07
FAM101B	2.46E-08	1.11E-09
DSEL	4.43E-05	5.73E-06
S100A4	8.05E-07	5.93E-08
SMAD7	2.43E-10	5.77E-12
RIIAD1	0.000594	0.000112
SERTAD1	7.00E-08	3.66E-09
MAGI2	9.73E-07	7.41E-08
SCUBE2	0.00908	0.00268
INSC	3.09E-06	2.74E-07
C10orf67	4.41E-07	3.00E-08
TLE2	8.30E-05	1.17E-05
GPRC5A	9.10E-10	2.64E-11
RYR2	5.16E-06	4.92E-07
GGT2	5.22E-05	6.90E-06
CLIP2	5.90E-05	7.95E-06
ARHGAP18	2.18E-08	9.63E-10
ENAH	5.31E-08	2.67E-09
EDNRA	4.87E-09	1.75E-10
C2CD4B	0.00803	0.00232
CTNNA1	1.13E-08	4.58E-10
DIAPH2	1.86E-09	5.91E-11
ENY2	1.83E-07	1.11E-08
TAPT1	2.10E-07	1.29E-08
TOM1L2	5.35E-12	7.64E-14
CRTAP	5.57E-10	1.49E-11
GPRASP1	4.49E-06	4.20E-07
TRIM58	1.47E-05	1.63E-06
EFEMP1	3.11E-08	1.45E-09
GSK3B	9.92E-13	1.13E-14

TLR8	0.00105	0.000217
LOC101928	1.61E-05	1.80E-06
BNIP2	2.64E-11	4.61E-13
MAGI1	1.09E-10	2.31E-12
CARMN	0.00105	0.000216
HMCN1	4.90E-05	6.43E-06
RASSF2	6.27E-08	3.23E-09
APOLD1	0.000577	0.000109
TMEM125	0.000329	5.69E-05
DOK2	2.71E-05	3.27E-06
AK7	0.000415	7.44E-05
EPN2-IT1	1.24E-07	7.09E-09
JMJD6	1.54E-09	4.80E-11
FAM122A	2.20E-13	1.99E-15
DIXDC1	1.15E-10	2.45E-12
CALHM2	3.38E-07	2.21E-08
MCAM	3.16E-08	1.48E-09
MT1F	1.08E-05	1.14E-06
HRCT1	0.000586	0.000111
SYNE3	6.08E-10	1.66E-11
FAM110D	0.000122	1.83E-05
RAB3IP	0.00193	0.000437
FRMD4B	9.75E-10	2.86E-11
RBM24	0.000777	0.000153
P2RY12	0.00028	4.73E-05
LOC101928	0.0011	0.000228
TTC25	0.00112	0.000234
DSTN	9.09E-11	1.87E-12
NATD1	1.34E-08	5.53E-10
RPA4	3.28E-06	2.93E-07
MYOZ1	2.88E-05	3.50E-06
KANSL1L	3.25E-11	5.81E-13
BCAR1	3.49E-05	4.36E-06
CITED2	8.24E-07	6.11E-08
TENM1	0.0074	0.00211
GJA4	6.92E-07	4.99E-08
MFNG	1.27E-07	7.25E-09
HDGFRP3	1.84E-06	1.53E-07
PRKCZ	6.39E-10	1.76E-11
EFR3B	4.34E-09	1.55E-10
SERPINI2	4.06E-05	5.19E-06
ALS2CL	2.19E-10	5.14E-12
ALOX5AP	8.56E-05	1.22E-05

FCAR	0.00681	0.00191
HSPB3	3.45E-05	4.30E-06
JDP2	3.38E-10	8.40E-12
MIR6756	2.52E-07	1.58E-08
NPY1R	0.000269	4.53E-05
CD69	0.000227	3.73E-05
GMFG	3.13E-05	3.85E-06
LOC101928	0.000393	6.98E-05
LST1	0.00018	2.85E-05
DOCK9	4.26E-10	1.10E-11
ZBTB46	1.47E-08	6.16E-10
STON1	1.66E-05	1.87E-06
ZC3H12C	2.50E-10	6.00E-12
DACT3	6.00E-07	4.25E-08
ARFGEF3	0.000126	1.89E-05
EFNB2	3.73E-07	2.48E-08
AK9	7.80E-05	1.09E-05
RORA	7.50E-08	3.97E-09
CHST3	1.22E-05	1.31E-06
BACH2	1.34E-08	5.52E-10
TJP1	6.40E-16	2.49E-18
SGCB	3.40E-09	1.17E-10
GPR153	4.04E-09	1.43E-10
KCNRG	0.000206	3.33E-05
ANXA11	4.72E-10	1.24E-11
HTT	3.71E-11	6.78E-13
PPFIBP1	1.72E-11	2.81E-13
PLPPR4	0.00443	0.00115
DDX5	4.33E-10	1.12E-11
FAM110B	4.32E-05	5.57E-06
PRUNE2	0.00855	0.0025
MEG3	0.0035	0.000875
CCDC173	0.000288	4.90E-05
DMRT2	0.000513	9.49E-05
CDC37L1	2.87E-09	9.59E-11
NDN	2.63E-06	2.29E-07
RALGAPA2	3.29E-05	4.07E-06
SLC12A4	3.55E-12	4.75E-14
DOCK6	1.04E-09	3.07E-11
GLT1D1	0.00014	2.13E-05
MINOS1	2.85E-08	1.32E-09
ZNF704	3.00E-05	3.66E-06
C1orf198	3.41E-13	3.26E-15

ARMC3	0.00492	0.0013
SLK	1.17E-12	1.36E-14
FBXO9	2.27E-06	1.94E-07
TMEM64	3.01E-08	1.40E-09
PXDC1	2.48E-11	4.29E-13
PDE3B	6.62E-07	4.75E-08
LCP2	0.000411	7.36E-05
MICU3	2.52E-06	2.19E-07
CD33	0.00162	0.000356
LGI4	2.24E-06	1.91E-07
PKN3	1.41E-06	1.12E-07
LOC101927	0.000172	2.70E-05
SLC8A1	2.40E-06	2.07E-07
C8B	0.00237	0.000554
DNAJB4	4.05E-08	1.97E-09
TMPRSS2	3.79E-05	4.78E-06
GNAQ	4.34E-14	3.16E-16
C8orf34	2.08E-05	2.41E-06
FAM124B	5.60E-06	5.38E-07
CASS4	5.16E-06	4.91E-07
SAP30L	1.13E-10	2.40E-12
MECP2	1.81E-09	5.71E-11
TNC	0.000829	0.000165
CALM3C	2.07E-18	2.20E-21
LRCH1	5.58E-11	1.08E-12
LOC101930	8.21E-08	4.41E-09
SLC4A4	2.81E-05	3.40E-06
APOL3	1.16E-07	6.58E-09
LINC00597	1.13E-08	4.59E-10
EOGT	5.81E-09	2.15E-10
VAMP2	1.17E-05	1.25E-06
NPDC1	5.85E-06	5.66E-07
ARHGAP23	6.75E-07	4.85E-08
TLR4	0.00015	2.31E-05
SPTLC3	0.00132	0.000283
SNCA	2.32E-08	1.04E-09
FAM105A	1.80E-05	2.04E-06
NCK1	5.89E-10	1.59E-11
SLPI	0.000113	1.67E-05
TNFRSF19	0.00158	0.000347
RGS22	1.54E-05	1.71E-06
S1PR2	7.59E-09	2.93E-10
GALNT13	0.000316	5.43E-05

MYO10	2.19E-08	9.68E-10
VLDLR	3.03E-05	3.69E-06
ARL13B	1.97E-10	4.54E-12
PLK3	7.99E-07	5.88E-08
BTG1	9.71E-08	5.36E-09
CCDC180	3.93E-05	4.99E-06
LOC101927	9.04E-06	9.33E-07
MOB3B	3.73E-07	2.49E-08
NDFIP2	9.20E-11	1.90E-12
HSDL2	1.07E-08	4.27E-10
NFIB	5.78E-09	2.14E-10
ADAMTS9-A	6.59E-05	9.04E-06
EVA1C	9.23E-06	9.57E-07
CPPED1	4.05E-07	2.72E-08
ARMC2	6.56E-06	6.46E-07
NINJ2	0.000175	2.75E-05
PROS1	1.89E-08	8.19E-10
MID1IP1	6.05E-06	5.87E-07
C8orf58	1.12E-08	4.51E-10
ANKRD33B	1.31E-06	1.04E-07
NEDD4	5.62E-07	3.94E-08
EGR2	0.000275	4.63E-05
CAMP	0.000124	1.85E-05
PDS5B	1.33E-11	2.08E-13
KCNS3	0.000336	5.84E-05
PDZRN4	3.89E-07	2.61E-08
PFKFB2	8.98E-05	1.29E-05
ADPRH	2.38E-09	7.78E-11
PSMA5	8.89E-06	9.16E-07
RSPH10B2	0.00208	0.000477
PRKCDBP	0.000105	1.54E-05
LOC729970	3.22E-07	2.10E-08
MAP6	2.22E-05	2.60E-06
GAB1	1.48E-08	6.20E-10
STX12	5.30E-14	3.96E-16
LOC153577	1.18E-06	9.25E-08
ARHGEF17	1.89E-07	1.15E-08
KLF11	1.22E-12	1.44E-14
GUCY1A3	7.72E-08	4.10E-09
DMPK	3.03E-05	3.69E-06
TLR3	3.54E-08	1.69E-09
TBC1D15	3.88E-09	1.36E-10
PIH1D3	0.00365	0.000917

AGPAT4-IT	0.000291	4.95E-05
INPP1	8.37E-11	1.70E-12
NBEAL1	5.98E-08	3.06E-09
INMT-FAM1	4.09E-07	2.75E-08
GJC1	2.96E-05	3.61E-06
PIK3R1	8.70E-08	4.72E-09
EPB41L4A	7.95E-06	8.06E-07
CSRP1	1.95E-09	6.23E-11
PRKCH	4.51E-12	6.26E-14
MAP3K8	1.86E-05	2.12E-06
TMEM232	0.00135	0.000288
DMTN	8.18E-06	8.35E-07
SGMS2	1.28E-05	1.39E-06
WBP11	2.26E-10	5.31E-12
IRF1	9.88E-06	1.03E-06
HSPA2	5.69E-07	4.00E-08
COX7B	1.00E-07	5.56E-09
TUBB6	4.81E-07	3.29E-08
STOM	1.68E-12	2.05E-14
MS4A7	0.000393	6.99E-05
GPSM1	1.50E-09	4.64E-11
LOC100507	0.00348	0.000869
WWC3	8.71E-12	1.30E-13
ZNF331	0.000211	3.43E-05
C11orf63	8.84E-05	1.26E-05
KLF13	5.10E-10	1.35E-11
NAPSB	0.00433	0.00112
AGPAT4	0.000375	6.62E-05
CLIC2	4.67E-06	4.39E-07
DNAH7	0.00101	0.000207
NIPAL3	5.04E-08	2.52E-09
TARPTR	0.000308	5.29E-05
ANO1	0.00139	0.000299
MYO1C	5.39E-11	1.03E-12
LRRFIP1	1.09E-10	2.31E-12
INPP5K	4.70E-13	4.71E-15
RERGL	0.000156	2.41E-05
RAB7B	0.00136	0.000292
CCDC114	0.0016	0.000353
ANKS1A	1.74E-12	2.14E-14
FAM13A-AS	1.59E-06	1.29E-07
SPECC1L-A	0.000123	1.84E-05
SLC15A2	2.48E-05	2.95E-06

MAST4	4.63E-07	3.15E-08
NPR2	1.76E-06	1.45E-07
MT1G	2.29E-05	2.69E-06
LNP1	4.03E-08	1.95E-09
SDR16C5	0.000855	0.000171
BTNL8	0.000256	4.27E-05
FZD1	1.99E-05	2.29E-06
RBM17	2.35E-15	1.18E-17
WBSCR17	7.99E-08	4.27E-09
PARD6B	1.17E-07	6.61E-09
GPR27	0.00195	0.000442
G0S2	0.00153	0.000334
BMP4	0.00106	0.000219
OCLN	0.000115	1.71E-05
C15orf52	2.52E-06	2.19E-07
SLC19A3	0.00453	0.00118
CRTAM	0.00813	0.00235
LONRF3	0.000177	2.80E-05
SOD3	0.0018	0.000404
STK17A	5.71E-05	7.67E-06
ABLIM1	6.88E-07	4.96E-08
PAPSS2	1.69E-12	2.07E-14
UNC13B	7.19E-06	7.18E-07
MVB12B	2.24E-07	1.39E-08
SMURF2	8.13E-08	4.36E-09
MSMO1	2.69E-07	1.70E-08
LOC154761	0.00404	0.00103
RFX3	1.47E-06	1.18E-07
FBXL7	2.89E-07	1.85E-08
COLEC12	8.57E-05	1.22E-05
SUGT1	4.25E-09	1.51E-10
EEF1A1	7.34E-07	5.33E-08
SECISBP2L	1.84E-09	5.84E-11
FFAR4	0.000386	6.83E-05
NDST1	6.91E-11	1.37E-12
SLC34A2	0.00722	0.00205
CD47	2.11E-09	6.80E-11
CDC42EP4	3.34E-10	8.27E-12
ARRDC4	2.84E-05	3.44E-06
ITPKC	0.00125	0.000265
RNASE4	9.16E-05	1.32E-05
USP53	5.92E-09	2.20E-10
ARMC4	0.007	0.00197

CCDC69	1.23E-07	7.05E-09
PF4	0.000126	1.89E-05
NUDT4	1.93E-05	2.22E-06
GABARAPL1	4.07E-09	1.44E-10
SEC63	3.24E-13	3.06E-15
JAG1	0.000963	0.000195
FGFR1	1.33E-06	1.05E-07
AKAP13	6.54E-08	3.39E-09
STX3	2.98E-12	3.90E-14
SPTAN1	6.99E-08	3.66E-09
CYB5A	1.40E-08	5.80E-10
ITPR1	2.56E-08	1.16E-09
FIP1L1	8.21E-08	4.42E-09
NFKBIA	6.11E-10	1.67E-11
TPR	3.28E-08	1.54E-09
SLC44A2	4.49E-10	1.16E-11
PER3	0.000753	0.000148
CD300LF	0.00132	0.000281
IRAK3	1.11E-05	1.18E-06
AQR	2.45E-10	5.84E-12
MRAS	4.08E-06	3.75E-07
NAP1L2	6.70E-05	9.21E-06
MPDZ	1.40E-06	1.12E-07
C15orf59	2.57E-06	2.23E-07
NRXN3	0.00175	0.00039
PCGF2	6.78E-06	6.71E-07
CPM	0.000109	1.60E-05
RGS18	0.0019	0.00043
BANCR	0.0023	0.000536
HIP1	8.88E-07	6.67E-08
NR2F2-AS1	2.53E-05	3.01E-06
PDE7B	0.00195	0.000443
EIF3F	1.06E-07	5.94E-09
WLS	2.99E-06	2.64E-07
TIAM1	1.22E-05	1.31E-06
DUSP6	2.95E-05	3.59E-06
LMO4	1.21E-05	1.30E-06
LOC728196	0.00101	0.000205
LOC101930	0.0073	0.00207
MNDA	0.000141	2.14E-05
LOC101929	0.00273	0.000654
TRIM36	0.000434	7.85E-05
SYNC	1.26E-07	7.24E-09

SPON1	0.00132	0.00028
ARMCX1	8.30E-07	6.16E-08
TNFSF12-T	1.33E-08	5.46E-10
HYMAI	1.79E-05	2.03E-06
EXT1	8.04E-08	4.30E-09
GJD3	3.52E-06	3.18E-07
KLF10	3.28E-08	1.54E-09
TDRD10	7.40E-06	7.42E-07
HOPX	0.000588	0.000111
CXCL12	0.000786	0.000155
PLEKHG1	1.00E-06	7.69E-08
SPI1	0.00314	0.000772
TM6SF1	3.68E-05	4.62E-06
PPM1F	1.20E-09	3.62E-11
MT1X	1.86E-05	2.12E-06
VNN2	0.00181	0.000406
ACTR3	1.97E-09	6.31E-11
PRKCB	0.000726	0.000141
AGTPBP1	2.69E-12	3.48E-14
KHDRBS2	3.29E-10	8.11E-12
C1orf162	2.00E-04	3.22E-05
MAGI3	7.25E-06	7.24E-07
LAMB2	2.31E-08	1.03E-09
ZC3H14	2.75E-07	1.75E-08
SGCA	3.42E-06	3.07E-07
LOC101930	0.000747	0.000146
TNFSF10	7.93E-06	8.04E-07
LOC101926	0.000192	3.07E-05
PKI55	2.39E-06	2.06E-07
UBL3	1.22E-10	2.61E-12
SLCO3A1	1.06E-07	5.89E-09
GYPC	9.16E-07	6.90E-08
WWTR1	9.63E-13	1.09E-14
AIMP1	5.60E-09	2.06E-10
MYO5B	9.26E-06	9.60E-07
CD109	0.00044	7.96E-05
CTNNBIP1	2.73E-06	2.39E-07
EPHA3	0.00592	0.00162
TFPI	4.08E-06	3.75E-07
SPN	6.67E-06	6.60E-07
ZBTB22	4.59E-06	4.31E-07
FLJ34503	0.00188	0.000425
TSPAN2	5.64E-07	3.96E-08

JUNB	5.57E-05	7.44E-06
UTRN	1.47E-12	1.78E-14
P2RY13	0.00225	0.000522
ALOX15	0.00269	0.000642
TCF4	6.90E-09	2.62E-10
TNFAIP1	1.06E-13	8.78E-16
PKDCC	0.000115	1.71E-05
RPGR	9.13E-08	4.99E-09
CDC42EP2	9.01E-09	3.55E-10
IL17RA	2.26E-08	1.01E-09
VAMP5	0.000449	8.15E-05
HACD4	2.44E-07	1.52E-08
LYRM9	1.32E-06	1.04E-07
CTDSPL	5.15E-09	1.87E-10
ARAP2	2.27E-08	1.01E-09
PPIL6	3.16E-09	1.07E-10
LMNA	6.61E-07	4.73E-08
MAP3K3	2.12E-11	3.59E-13
DHCR24	4.23E-05	5.43E-06
C20orf194	3.51E-08	1.67E-09
ITPKB	1.89E-07	1.15E-08
ITGA5	4.00E-06	3.67E-07
PDCD2	7.12E-07	5.15E-08
DNAJC27	5.74E-10	1.54E-11
VEGFC	1.79E-05	2.03E-06
METTL7A	1.59E-06	1.29E-07
CSRP2	6.36E-07	4.53E-08
SERP2	2.00E-05	2.31E-06
TTC29	0.00298	0.000724
BCAP29	7.73E-08	4.11E-09
WDR63	0.00657	0.00183
NFATC4	0.000287	4.87E-05
SCAMP1	1.34E-08	5.53E-10
DGKH	1.92E-08	8.40E-10
MAMLD1	0.00191	0.000433
GTDC1	7.52E-12	1.10E-13
MTMR10	2.35E-09	7.68E-11
ZDHHC11	0.000235	3.87E-05
EDIL3	5.14E-05	6.78E-06
RIN2	1.88E-05	2.15E-06
MAN2A2	4.51E-09	1.61E-10
TWIST2	0.00137	0.000295
ANXA1	1.54E-06	1.24E-07

MOB3C	3.65E-08	1.75E-09
HNRNPA0	7.28E-11	1.46E-12
DDR2	1.93E-06	1.62E-07
PTGS1	1.48E-05	1.63E-06
MSR1	0.00121	0.000255
TMEM71	0.00315	0.000773
ACSL4	0.000382	6.75E-05
FLT1	9.84E-08	5.44E-09
TTC7B	2.35E-09	7.69E-11
A2M	5.21E-09	1.89E-10
FAM171A1	0.000306	5.25E-05
RASSF8-AS	7.55E-06	7.59E-07
BIRC3	0.00117	0.000245
CCDC181	0.000998	0.000203
ABCC9	1.07E-05	1.13E-06
LURAP1L	5.76E-06	5.56E-07
ZSCAN18	2.17E-05	2.54E-06
DOT1L	5.65E-05	7.57E-06
USP7	3.54E-10	8.86E-12
CYP2U1	1.23E-07	6.99E-09
ZAK	1.59E-08	6.76E-10
MAP7D3	1.81E-09	5.74E-11
APLN	0.000693	0.000134
ABCG1	0.000347	6.05E-05
CCDC96	3.82E-05	4.83E-06
SOCS2-AS1	1.90E-06	1.59E-07
LAMTOR3	5.51E-11	1.06E-12
PARD3	2.53E-07	1.59E-08
LCA5	0.000545	0.000102
GTF2IP12	5.76E-07	4.05E-08
TBC1D24	0.00625	0.00172
PPP1R3E	2.60E-05	3.12E-06
ELL3	3.47E-06	3.13E-07
RPGRIP1L	0.000104	1.51E-05
AP3D1	2.65E-05	3.18E-06
SMG7	1.87E-06	1.56E-07
CNIH4	1.74E-06	1.44E-07
STT3A	2.70E-11	4.73E-13
PEX13	3.75E-07	2.50E-08
NME4	4.82E-07	3.30E-08
SMC2	6.77E-05	9.32E-06
LRIG3	0.000128	1.93E-05
MIR181A2H	3.95E-05	5.03E-06

FGFR1OP2	1.28E-05	1.39E-06
AHCTF1P1	3.62E-06	3.28E-07
CNPY2	1.30E-10	2.80E-12
ZSCAN2	7.05E-09	2.69E-10
LOC728613	1.01E-05	1.06E-06
BDH1	0.00212	0.000487
POLG2	1.90E-10	4.35E-12
OLMALINC	7.79E-06	7.87E-07
FLJ32255	0.000672	0.000129
HILPDA	0.0079	0.00227
DAPP1	4.68E-05	6.10E-06
TIPIN	3.82E-06	3.49E-07
DDIT4	6.49E-05	8.89E-06
LOC100507	0.00894	0.00263
ESRP1	0.00125	0.000264
MROH6	8.30E-05	1.17E-05
PLGLB1	0.00239	0.00056
TOR3A	1.14E-11	1.76E-13
ACVR1C	0.00115	0.000239
PRKAB2	1.07E-05	1.13E-06
CBX3	7.20E-10	2.02E-11
RPL17-C18	7.40E-05	1.03E-05
SSX2IP	5.92E-06	5.73E-07
SLC39A11	8.38E-07	6.23E-08
FAM114A1	1.48E-06	1.19E-07
CYAT1	0.00295	0.000716
CCDC18	1.74E-05	1.97E-06
RIMS2	0.002	0.000456
SLC25A15	0.000117	1.73E-05
MTFR1	8.48E-08	4.58E-09
MIR6834	2.06E-08	9.06E-10
CEP152	5.50E-09	2.02E-10
C4orf46	3.30E-05	4.09E-06
SLC39A4	0.00255	0.000604
RMI1	9.06E-08	4.94E-09
LOC101927	5.59E-10	1.49E-11
ATG16L1	1.06E-06	8.18E-08
EAF2	1.33E-06	1.06E-07
TSNAX-DIS	0.000477	8.73E-05
LAMP5	0.00611	0.00168
INTS8	1.73E-10	3.92E-12
TCEA1	1.55E-10	3.45E-12
PGF	0.000173	2.72E-05

GOLGA7B	0.00877	0.00257
RAPH1	0.000231	3.79E-05
ARL14	0.0089	0.00261
HSD3B7	6.27E-05	8.52E-06
CAPN5	0.00119	0.00025
DIEXF	1.78E-11	2.93E-13
KRT15	0.00504	0.00134
HEXIM2	1.00E-07	5.57E-09
WDR59	9.35E-07	7.07E-08
PSD3	0.00129	0.000273
CSTF3	7.21E-09	2.76E-10
FAM213A	2.17E-07	1.34E-08
ZNF850	0.000134	2.02E-05
C6orf141	0.00838	0.00243
FGFR1OP	1.06E-08	4.24E-10
DNAJC3	5.37E-08	2.71E-09
OVOL1	1.50E-05	1.66E-06
ZDHHC13	1.99E-10	4.60E-12
GTF3C3	1.31E-05	1.43E-06
MMD	0.000343	5.97E-05
SLC38A9	4.51E-07	3.07E-08
MRPS12	2.03E-06	1.71E-07
MTFP1	2.54E-05	3.04E-06
RFC5	2.02E-06	1.70E-07
NMNAT2	0.000679	0.000131
37500	5.43E-07	3.78E-08
OSMR	0.00441	0.00114
ANXA9	0.00364	0.000915
SNX27	2.77E-08	1.28E-09
LILRB4	0.00577	0.00157
TIMM50	5.63E-07	3.95E-08
RSRC1	8.42E-07	6.28E-08
LOC101929	0.00999	0.003
YME1L1	4.59E-06	4.31E-07
METTL8	3.14E-10	7.73E-12
WHSC1	1.18E-06	9.24E-08
SMKR1	0.00636	0.00176
RALY-AS1	5.43E-05	7.22E-06
MIR210HG	0.00101	0.000206
SLC39A7	2.15E-06	1.83E-07
TACC3	1.69E-08	7.21E-10
GMPS	5.51E-10	1.47E-11
HS3ST1	0.00453	0.00118

DNAJC3-AS	4.19E-06	3.87E-07
CDC14B	7.94E-05	1.12E-05
ALG3	4.97E-09	1.79E-10
SORL1	4.36E-05	5.62E-06
MCRIP2	3.25E-06	2.91E-07
DENND1B	2.18E-05	2.55E-06
METTL26	0.000177	2.79E-05
PPFIA1	2.11E-10	4.91E-12
PC	1.16E-05	1.24E-06
ZNF883	0.00271	0.000649
SNHG1S	2.42E-09	7.92E-11
EPHX4	0.000185	2.94E-05
RARRES1	0.000695	0.000134
E2F5	5.72E-07	4.02E-08
CCL7	0.00263	0.000626
RBM12B	1.04E-08	4.16E-10
PRR5-ARHG	0.000337	5.86E-05
NSD1	8.77E-11	1.79E-12
PPIL1	7.57E-08	4.01E-09
CHAC2	3.50E-05	4.38E-06
TNFRSF9	0.00203	0.000465
CHEK2	1.79E-06	1.48E-07
OLFML2B	8.25E-05	1.17E-05
SDCCAG8	4.38E-09	1.56E-10
PPP4R3B	4.58E-06	4.30E-07
IKBKE	8.80E-08	4.78E-09
FUT6	0.000158	2.45E-05
CTSK	0.000111	1.64E-05
GPR84	0.00446	0.00116
PUS7	2.40E-08	1.08E-09
ATP2A2	1.46E-10	3.21E-12
MUC3B	0.0029	0.000701
DUSP4	0.000235	3.87E-05
FOLR1	2.42E-09	7.92E-11
TIMP1	6.65E-09	2.51E-10
HOXC9	0.0041	0.00105
ARHGEF16	3.66E-05	4.60E-06
DNPH1	4.31E-06	4.00E-07
TMED3	6.49E-09	2.44E-10
LOC101929	4.94E-06	4.68E-07
LOC101928	0.0025	0.000589
FAM60A	1.87E-06	1.56E-07
LOC374443	3.50E-05	4.38E-06

TRAF4	1.87E-06	1.56E-07
LOC100506	0.00128	0.000272
MRPS33	4.13E-09	1.46E-10
LOC101930	9.04E-06	9.33E-07
CUL7	3.19E-05	3.94E-06
COL7A1	4.87E-05	6.37E-06
PCNA	3.91E-07	2.62E-08
FLAD1	7.03E-08	3.68E-09
PKM	9.20E-06	9.52E-07
FAM24B	8.87E-08	4.83E-09
SUGCT	1.86E-05	2.13E-06
B4GALT4	1.34E-08	5.51E-10
KIF2A	1.86E-09	5.91E-11
SRPK1	1.58E-10	3.53E-12
UBE2QL1	8.45E-06	8.64E-07
MFSD9	2.69E-09	8.94E-11
HN1L	9.34E-09	3.69E-10
PNKD	2.13E-05	2.47E-06
KDM5B	1.80E-10	4.09E-12
LOC100131	0.00103	0.000211
TTC22	0.000271	4.57E-05
PAIP1	2.33E-08	1.04E-09
GPR78C	0.00111	0.000231
MGST1	0.00214	0.000493
HEATR3	2.14E-08	9.44E-10
ZNF260	2.05E-07	1.25E-08
XPO5	1.62E-07	9.62E-09
EXOC7	2.83E-06	2.49E-07
BRCA1	7.29E-06	7.29E-07
ATIC	1.49E-10	3.29E-12
CHD4	1.33E-05	1.45E-06
ZNF738	4.12E-06	3.80E-07
SSR4	4.54E-13	4.54E-15
DMXL2	0.000167	2.60E-05
LOC100133	0.000632	0.000121
UBE2S	0.000242	4.00E-05
SRM	5.67E-08	2.89E-09
KRTCAP3	0.000546	0.000102
ATP11B	7.36E-08	3.88E-09
CCDC66	1.84E-08	7.94E-10
MANF	1.73E-13	1.53E-15
IGFBP5	2.83E-05	3.43E-06
FAM64A	0.00338	0.000839

LOC100287	1.02E-13	8.32E-16
FAM53A	4.40E-06	4.10E-07
MLLT11	0.00538	0.00144
PRLR	0.00863	0.00252
SOX12	2.69E-07	1.70E-08
MIF	2.81E-13	2.63E-15
IDH2	1.67E-09	5.22E-11
COL15A1	0.000188	2.99E-05
MAPK13	3.04E-07	1.95E-08
PSPH	1.92E-06	1.61E-07
GRHL1	0.0052	0.00139
STARD5	5.51E-05	7.34E-06
MAP2K6	0.00103	0.000212
TRIM14	4.84E-06	4.58E-07
CKS2	2.94E-05	3.57E-06
EPM2AIP1	0.00229	0.000533
ANO9	5.58E-05	7.46E-06
C1QTNF6	6.38E-08	3.30E-09
KBTBD12	0.00103	0.000211
CBX5	3.62E-05	4.55E-06
TTYH3	4.01E-07	2.69E-08
SNORA72	9.23E-08	5.06E-09
C1GALT1	6.02E-08	3.09E-09
H1F0	1.34E-05	1.47E-06
SEMA4B	2.16E-06	1.84E-07
FANCG	5.67E-11	1.10E-12
HPS3	3.91E-17	1.01E-19
LOC101929	0.00218	0.000504
ELOVL6	0.00112	0.000233
PDLIM4	0.000225	3.67E-05
ZC3H8	2.33E-08	1.04E-09
SLC25A10	1.63E-05	1.83E-06
ADAM8	1.97E-05	2.27E-06
MMP16	0.0018	0.000404
RAB30-AS1	7.76E-05	1.09E-05
PLTP	0.000237	3.91E-05
BRCA2	1.90E-07	1.15E-08
HOXC4	2.75E-07	1.75E-08
BOLA2B	7.59E-09	2.93E-10
BZW2	1.59E-09	4.95E-11
ATAD5	8.14E-07	6.01E-08
LY9	0.00154	0.000336
SNORD88C	6.67E-06	6.59E-07

SLC38A10	1.37E-06	1.09E-07
PIGM	1.71E-07	1.02E-08
IL11	0.00572	0.00155
DUS4L	3.08E-07	1.98E-08
GPR180	2.02E-11	3.38E-13
MMP3	0.000545	0.000102
LINC00847	4.93E-08	2.46E-09
CAD	7.95E-09	3.09E-10
RNFT2	0.00162	0.000358
IGLV6-57	1.58E-05	1.76E-06
DHTKD1	3.22E-09	1.10E-10
TKT	0.00523	0.0014
CYP2C18	0.00767	0.0022
EPHA4	0.00355	0.000889
SLC22A18	3.02E-06	2.67E-07
LOC100272	6.08E-07	4.31E-08
SCG2	0.00545	0.00147
FAAH2	2.15E-06	1.82E-07
PLEKHA6	0.00082	0.000163
DNMT3A	8.22E-08	4.42E-09
LOC100288	1.68E-06	1.38E-07
NSUN5P1	1.21E-09	3.66E-11
DONSON	1.38E-07	7.98E-09
TRAPPC10	5.06E-07	3.49E-08
HSPA13	3.49E-06	3.14E-07
PLEKHA8	3.46E-11	6.25E-13
TCF19	1.02E-05	1.07E-06
NCAPD2	4.25E-07	2.87E-08
LYPD1	0.00399	0.00102
PSMG3	2.37E-08	1.07E-09
SPDL1	1.58E-06	1.28E-07
ITM2C	5.50E-09	2.02E-10
HCG8ZN	0.000317	5.46E-05
CKS1B	1.02E-05	1.07E-06
LYPD3	0.00157	0.000343
CCDC80	0.0038	0.000961
SMIM22	7.96E-06	8.08E-07
PLVAP	0.000137	2.09E-05
NUP155	6.88E-07	4.96E-08
LINC01355	6.80E-09	2.57E-10
TET1	0.000107	1.57E-05
RPL37A	2.26E-05	2.65E-06
NIPSNAP1	2.31E-08	1.03E-09

TRIM11	1.42E-06	1.14E-07
TRMT13	2.00E-05	2.30E-06
SLC1A4	3.60E-08	1.72E-09
NPM1	1.52E-08	6.43E-10
SHOX2	0.00438	0.00114
MOCOS	0.00264	0.000627
MSH5-SAPC	1.37E-06	1.09E-07
FANCF	3.98E-06	3.65E-07
HNF4G	0.00129	0.000273
GXYLT2	1.42E-05	1.55E-06
C1orf43	2.24E-05	2.62E-06
SUSD4	0.00483	0.00127
COX15	5.59E-11	1.08E-12
EME1	9.84E-06	1.03E-06
LOC441666	0.000414	7.41E-05
DNA2	7.76E-08	4.13E-09
LEF1	1.65E-06	1.35E-07
YPEL3	0.00067	0.000129
KLHL7	2.18E-05	2.55E-06
RHNO1	1.91E-09	6.08E-11
THRILB	1.79E-08	7.73E-10
SNRNP25	3.20E-09	1.09E-10
TRIB3	0.000218	3.54E-05
IPO4	4.42E-07	3.00E-08
ABHD11	2.14E-05	2.49E-06
HIST1H2AM	9.11E-05	1.31E-05
CDK16	1.05E-07	5.84E-09
OAZ3	0.000159	2.47E-05
CMBL	0.00434	0.00112
SEMA3A	0.00572	0.00155
ABCB6	5.84E-06	5.64E-07
SARS2	5.78E-07	4.07E-08
NAV1	3.17E-06	2.82E-07
VARS	7.63E-08	4.05E-09
SLC7A1	1.92E-05	2.20E-06
RRBP1	0.000101	1.48E-05
ELMOD2	1.44E-09	4.44E-11
FBXL20	3.38E-07	2.21E-08
SNORD77	7.20E-10	2.02E-11
EPRS	1.13E-09	3.38E-11
POF1B	0.00442	0.00115
KIAA1211	0.000968	0.000196
DBNDD1	0.000479	8.79E-05

TTC9	0.000599	0.000113
PAK6	0.000254	4.23E-05
MGC57346	2.37E-06	2.04E-07
DLG3	4.06E-05	5.19E-06
SSR3	1.69E-14	1.08E-16
L2HGDH	2.46E-05	2.94E-06
HOXB-AS1	0.000161	2.51E-05
SLC2A11	2.96E-06	2.62E-07
BMF	6.45E-07	4.60E-08
PARPBP	2.75E-06	2.41E-07
SLC4A11	0.000629	0.00012
PARS2	4.21E-06	3.90E-07
SEL1L3	1.66E-11	2.70E-13
LAGE3	1.77E-07	1.06E-08
CCDC167	1.77E-08	7.60E-10
MUC20	0.00702	0.00198
CENPM	2.36E-08	1.06E-09
STK31	0.00131	0.000279
CENPH	1.15E-05	1.22E-06
UBE2Z	4.85E-07	3.33E-08
F2RL1	4.00E-05	5.10E-06
MIR612	0.000233	3.84E-05
H2AFX	3.01E-09	1.02E-10
SLC29A2	0.00106	0.000218
GSDMB	2.10E-09	6.76E-11
LOC102723	0.00504	0.00134
HS3ST3A1	1.97E-05	2.27E-06
UQCC1	9.49E-10	2.77E-11
RAB15	0.000421	7.56E-05
ARFGEF2	6.91E-06	6.85E-07
SLFN13	5.36E-05	7.12E-06
FAR2P3	0.00928	0.00274
MYO19	4.52E-10	1.17E-11
PRR5	6.67E-07	4.79E-08
PTK6	5.64E-05	7.54E-06
BRI3BP	2.25E-08	1.00E-09
KIF21A	9.50E-06	9.88E-07
TRIM6	0.000197	3.17E-05
GDPD1	0.000473	8.65E-05
PLK1	0.00387	0.000983
DPY19L1	3.04E-05	3.71E-06
PMS2P5	1.38E-09	4.23E-11
CHML	0.000124	1.87E-05

GCAT	0.000236	3.89E-05
RNF207	3.29E-07	2.15E-08
YKT6	1.47E-06	1.18E-07
IL20RB	0.00802	0.00231
SNHG4M	0.000227	3.71E-05
SKP2	2.53E-07	1.59E-08
ST14	1.78E-06	1.47E-07
FAM46C	0.000418	7.50E-05
TBL1XR1	1.21E-09	3.65E-11
MIS18A	3.85E-07	2.57E-08
HIST1H2AH	0.000269	4.52E-05
SLC50A1	5.83E-10	1.58E-11
CHRDL2	0.000651	0.000125
STEAP3	6.37E-07	4.54E-08
ARHGEF19	9.96E-05	1.45E-05
MGA	7.73E-06	7.80E-07
MUC13	0.00679	0.0019
QPCT	0.00194	0.00044
SCUBE3	0.00169	0.000374
CEP41	1.36E-06	1.08E-07
AP1S1	3.38E-10	8.39E-12
SUZ12	5.07E-14	3.76E-16
ZC3H12D	1.27E-05	1.37E-06
PFKP	1.67E-07	9.95E-09
XRN1	6.72E-07	4.82E-08
SRPRB	2.71E-11	4.76E-13
SSBP1	1.51E-07	8.91E-09
MCCC2	1.71E-07	1.02E-08
HYLS1	5.92E-06	5.73E-07
MSH5-SAPC	5.00E-07	3.45E-08
CBFA2T2	1.31E-13	1.13E-15
MCM6	3.08E-10	7.54E-12
GFPT1	3.62E-09	1.26E-10
ZNF519	1.45E-05	1.60E-06
JMY	9.55E-08	5.25E-09
CYP2J2	0.00373	0.000942
IGHV1-69	0.000838	0.000167
STRBP	2.08E-12	2.62E-14
PLD1	1.49E-07	8.72E-09
PRR36	0.00231	0.000539
SGPL1	2.38E-17	5.48E-20
FAM83B	0.00254	6.00E-04
HDHD3	4.32E-05	5.57E-06

LOC389834	0.00139	0.000299
TMPO	3.21E-08	1.51E-09
MPZL1	5.37E-10	1.43E-11
PMS2P5	1.26E-10	2.72E-12
SMPDL3B	2.88E-09	9.65E-11
PSENEN	1.28E-06	1.01E-07
CYP1B1	0.000494	9.08E-05
CD180	4.41E-06	4.12E-07
LOC102724	0.00192	0.000435
HLA-DOB	0.000648	0.000124
UPK3BL	1.13E-06	8.78E-08
XPR1	1.06E-08	4.25E-10
GMDS	1.11E-06	8.60E-08
GAPDHP62	6.34E-08	3.28E-09
EHF	0.00268	0.000638
PIK3CB	1.02E-07	5.67E-09
TMEM156	1.20E-05	1.28E-06
CBLC	0.00306	0.000746
REEP6	0.000458	8.34E-05
LOC100996	0.000331	5.73E-05
CSMD2	2.06E-06	1.74E-07
OSBPL3	3.31E-09	1.13E-10
GPR35	0.0018	0.000402
XPO1	5.64E-10	1.51E-11
RCCD1	6.07E-10	1.65E-11
TBC1D31	4.30E-06	3.99E-07
HIST1H2AC	2.06E-05	2.38E-06
BPTF	2.06E-11	3.49E-13
ITCH	6.07E-09	2.26E-10
RNF183	0.00484	0.00127
TMEM33	7.19E-06	7.17E-07
RHOH	0.000785	0.000155
WNK1	9.27E-08	5.08E-09
TMEM106B	2.18E-11	3.71E-13
FKBP10	3.06E-06	2.71E-07
XPO7	4.42E-07	3.00E-08
ZNF692	5.13E-09	1.86E-10
SFXN4	5.81E-10	1.57E-11
SPCS3	7.07E-10	1.98E-11
GPI	2.86E-12	3.71E-14
CFB	1.64E-05	1.85E-06
ZNF793-AS	0.000102	1.49E-05
LOC100996	9.20E-08	5.03E-09

HHIPL2	0.00946	0.00281
PRKCA	0.000103	1.50E-05
SLC25A36	2.94E-07	1.88E-08
RACGAP1	1.48E-07	8.65E-09
ACP6	6.06E-07	4.29E-08
C1orf53	1.33E-05	1.45E-06
STK39	1.83E-07	1.10E-08
PKMYT1	6.95E-08	3.63E-09
RHOV	0.00103	0.000212
CAPN12	0.00101	0.000206
FAM98A	4.78E-08	2.37E-09
FCRL2	0.000539	1.00E-04
SAC3D1	7.15E-09	2.74E-10
OR7E14P	7.97E-05	1.12E-05
GAL	0.0048	0.00126
MLEC	9.15E-09	3.61E-10
LOC728613	6.83E-05	9.41E-06
PHKA1	2.00E-07	1.22E-08
TMTC4	5.05E-08	2.53E-09
PTGR1	3.34E-05	4.14E-06
TANC2	4.23E-06	3.92E-07
SYNJ2	1.52E-06	1.23E-07
GALNT6	0.00036	6.31E-05
KNSTRN	1.30E-07	7.50E-09
MCM8	2.61E-07	1.65E-08
DCBLD2	0.00027	4.54E-05
GIGYF2	4.41E-09	1.57E-10
TIGD1	7.84E-09	3.04E-10
RNASEH2A	5.95E-06	5.76E-07
LMNB1	4.57E-06	4.28E-07
PKIB	0.000509	9.40E-05
CREB3L4	2.26E-05	2.65E-06
ATL3	6.92E-08	3.61E-09
MPP6	8.64E-06	8.88E-07
MCM3AP-AS	3.33E-08	1.57E-09
EMC3-AS1	1.14E-06	8.83E-08
C12orf66	7.26E-06	7.26E-07
TNFRSF21	1.94E-08	8.49E-10
TFRC	2.93E-05	3.56E-06
SFXN1	9.49E-12	1.42E-13
SNHG3S	2.79E-06	2.45E-07
CDCP1	4.22E-05	5.42E-06
SDCCAG3	3.92E-10	9.97E-12

APELA	0.000867	0.000173
KDELR2	4.27E-12	5.89E-14
PRRT3-AS1	7.37E-06	7.39E-07
BCL11A	8.28E-07	6.14E-08
RNF139-AS	4.05E-10	1.03E-11
NEIL3	0.000926	0.000187
FAM69A	4.05E-10	1.04E-11
FBXO16	6.79E-07	4.89E-08
CCDC150	0.000325	5.63E-05
ZFAS1	1.81E-05	2.06E-06
FLJ35934	1.22E-07	6.96E-09
BARD1	6.38E-08	3.30E-09
GGCT	1.28E-11	1.99E-13
GPR89B	2.90E-09	9.73E-11
TCF3	3.95E-15	2.13E-17
DSG2	1.10E-05	1.17E-06
EPYC	0.00383	0.000971
DSCC1	5.83E-05	7.86E-06
VCAN	2.42E-06	2.09E-07
POPDC3	0.00713	0.00202
ATAT1	0.000203	3.28E-05
EIF4EBP1	2.96E-10	7.22E-12
LAX1	9.25E-05	1.33E-05
MIS18BP1	1.33E-06	1.06E-07
UROS	3.29E-07	2.15E-08
FOXA3	0.000426	7.68E-05
CBX2	0.00566	0.00153
H2BFS	1.24E-07	7.09E-09
NFATC2IP	1.01E-09	2.98E-11
MAPKAP1	1.03E-05	1.08E-06
NT5E	4.49E-06	4.20E-07
GLB1L2	0.00616	0.00169
CERCAM	3.95E-08	1.91E-09
WFDC2	0.00118	0.000247
LOC100129	8.24E-07	6.11E-08
LOC283177	0.000145	2.22E-05
PLA2G7	0.00134	0.000288
LPGAT1	3.09E-07	1.99E-08
ASPH	0.000895	0.00018
KIAA1524	4.75E-07	3.25E-08
LOC441155	2.46E-05	2.93E-06
SEC24D	4.72E-10	1.24E-11
SPIB	0.000186	2.96E-05

AHNAK2	0.000753	0.000148
CHRNA5	0.00464	0.00121
PKP2	0.00929	0.00275
GMPPA	3.16E-11	5.62E-13
ZBED6	1.80E-05	2.04E-06
ALDH1L2	9.78E-05	1.42E-05
HSH2D	2.60E-08	1.19E-09
SRD5A3	4.31E-07	2.92E-08
CEP72	5.15E-06	4.91E-07
ATG7	7.16E-07	5.18E-08
CENPW	4.15E-05	5.32E-06
HOOK1	1.62E-06	1.32E-07
NQO1	0.00641	0.00178
SYT1	0.00521	0.00139
ALPK2	0.00137	0.000294
HGS	1.08E-07	6.06E-09
IGF1	0.00587	0.0016
CTSV	0.00188	0.000424
EXOSC5	7.44E-06	7.47E-07
ZMYND8	7.33E-11	1.47E-12
WDR12	9.94E-10	2.92E-11
EPHX3	6.50E-06	6.38E-07
ZNF664-FA	0.000809	0.00016
GALNT7	1.91E-10	4.37E-12
CDC25C	0.00256	0.000607
TMPRSS11E	0.00845	0.00246
IGFL2	0.000256	4.26E-05
HIST1H2BC	0.00019	3.02E-05
NFE2L3	3.92E-08	1.89E-09
RBM15	5.41E-08	2.73E-09
PTGFRN	4.06E-12	5.53E-14
DDX11	9.97E-08	5.52E-09
TPD52	2.11E-13	1.89E-15
MMP14	1.30E-10	2.82E-12
LSR	1.32E-05	1.43E-06
GGH	4.07E-05	5.20E-06
PDCD2L	5.99E-06	5.80E-07
BBOX1-AS1	0.000843	0.000168
MARVELD3	3.54E-06	3.19E-07
CEACAM7	0.00767	0.0022
CLDN3	0.00422	0.00109
AIFM2	2.35E-05	2.78E-06
P2RX5	0.00159	0.000349

QSER1	1.30E-07	7.47E-09
FUT8	2.37E-13	2.17E-15
TMEM132A	6.32E-09	2.36E-10
ZNF750	0.00122	0.000257
PPP1R14B	7.02E-11	1.40E-12
HN1	3.33E-08	1.57E-09
RAB42	2.56E-06	2.22E-07
SHMT2	1.67E-12	2.03E-14
BEAN1	0.000295	5.02E-05
MIR7112	6.30E-07	4.48E-08
TPBG	1.72E-10	3.89E-12
ACE2	0.000207	3.35E-05
PDCD6	5.82E-10	1.57E-11
HIST1H3E	4.33E-06	4.02E-07
GLDC	0.00252	0.000594
TRABD	5.02E-07	3.46E-08
KIF18A	0.000103	1.51E-05
SYNGR3	3.13E-05	3.85E-06
SBK1	4.47E-05	5.80E-06
SLC16A9	0.00661	0.00184
HIST3H2A	3.00E-07	1.92E-08
ATP6V0A4	0.0031	0.000759
SOX4	1.19E-09	3.57E-11
CST1	0.0071	0.00201
DSP	0.000722	0.00014
NHS	4.12E-06	3.80E-07
PCBD2	3.36E-11	6.04E-13
SLC9A7	6.67E-10	1.85E-11
SORD	5.97E-07	4.22E-08
CPXM1	0.000439	7.94E-05
FANCA	1.75E-08	7.52E-10
HIST1H4H	6.66E-05	9.16E-06
UGGT1	9.05E-13	1.01E-14
ZMYM2	3.61E-08	1.72E-09
SNX5	5.82E-07	4.10E-08
SLC17A9	7.30E-09	2.81E-10
CLASP1	1.26E-07	7.18E-09
UBN2	2.96E-07	1.90E-08
RPP40	3.49E-06	3.14E-07
RPL7	3.61E-10	9.08E-12
PKP3	1.87E-05	2.14E-06
DSG2-AS1	0.000316	5.43E-05
FGF11	2.96E-05	3.61E-06

MET	0.00571	0.00155
ABCA12	0.00418	0.00107
TMEM177	2.40E-08	1.08E-09
LARGE2	0.000442	8.00E-05
FCRLA	0.000203	3.28E-05
UQCRC2	7.11E-09	2.72E-10
CASP2	7.48E-08	3.96E-09
MPHOSPH9	1.39E-11	2.20E-13
SUV39H2	3.49E-08	1.66E-09
LOX	0.000178	2.82E-05
BAIAP2L1	2.71E-07	1.72E-08
FAM199X	2.56E-10	6.16E-12
MARCKSL1	1.26E-10	2.71E-12
DTYMK	4.37E-10	1.13E-11
MTFR2	2.30E-05	2.71E-06
SYNE4	0.000612	0.000116
TNPO1	6.68E-15	3.85E-17
IGHV4-31	6.07E-06	5.90E-07
MIR6787	5.29E-08	2.66E-09
ZYG11A	0.00211	0.000485
NDC1	3.17E-08	1.48E-09
HMBS	2.86E-11	5.04E-13
LINC01138	1.62E-08	6.88E-10
NCAPH	0.000331	5.74E-05
RNF43	6.13E-05	8.30E-06
TUBB2B	0.000162	2.52E-05
EXO1	0.000229	3.75E-05
THRAP3	4.51E-08	2.22E-09
SULT1C2	0.00113	0.000235
LOXL2	3.46E-07	2.27E-08
RPL39L	2.07E-05	2.40E-06
PRRC1	2.37E-11	4.08E-13
CCL19	0.00669	0.00187
GALNT2	1.20E-09	3.63E-11
ADORA3	0.00307	0.000751
BICDL1	1.19E-07	6.76E-09
DHRS2	0.00925	0.00273
PHLDA2	0.000108	1.59E-05
TCERG1	6.72E-07	4.83E-08
STRIP2	0.000179	2.83E-05
ANKRD13D	1.03E-09	3.05E-11
TMCC1	6.59E-10	1.82E-11
SGPP2	4.00E-04	7.12E-05

MTA3	2.39E-10	5.66E-12
RECQL4	4.12E-05	5.27E-06
SDF2L1	4.95E-10	1.31E-11
RUNX2	6.47E-10	1.79E-11
TNFSF11	0.000111	1.64E-05
SAA2SA	0.00373	0.00094
HOXB9	0.00616	0.00169
HOXD10	0.00515	0.00137
BICD1	1.44E-05	1.59E-06
ATAD2	4.86E-08	2.42E-09
TMEM182	7.26E-10	2.05E-11
C1orf112	5.88E-07	4.15E-08
FAM173B	1.44E-09	4.42E-11
KIAA1324	0.00207	0.000474
CD24	9.94E-05	1.44E-05
FAM3C	2.91E-07	1.86E-08
NUP62CL	5.95E-05	8.03E-06
P2RY6	1.41E-07	8.25E-09
FKBP14	4.63E-12	6.46E-14
MMP13	0.00134	0.000287
PRDX4	8.08E-17	2.23E-19
FAM26F	0.00192	0.000435
KCNE4	0.000185	2.94E-05
UPK1B	0.00639	0.00177
LOC284926	1.18E-07	6.70E-09
PRIM1	6.59E-07	4.71E-08
PHF19	1.29E-07	7.40E-09
LINC00115	5.47E-11	1.05E-12
CERS6	1.49E-08	6.26E-10
FAM72A	2.11E-05	2.45E-06
ENTPD7	1.28E-08	5.25E-10
GNG4	0.00106	0.000219
KCNN3	4.57E-05	5.94E-06
RAD51	1.18E-07	6.65E-09
BLACAT1	0.00015	2.31E-05
STX1A	6.19E-07	4.40E-08
FIGNL1	9.56E-10	2.79E-11
KRT17J	0.000384	6.81E-05
FMO5	0.00214	0.000492
PIN4	3.16E-08	1.48E-09
LRP8	9.03E-07	6.80E-08
CHI3L1	2.09E-05	2.42E-06
C4orf48	1.05E-08	4.21E-10

FANCD2	5.63E-09	2.07E-10
GMNN	3.93E-10	1.00E-11
GTF2IP20	2.61E-06	2.27E-07
CEP128	1.77E-06	1.46E-07
LINC00467	5.60E-08	2.85E-09
MIR3934	7.19E-13	7.78E-15
ZNF215	6.34E-06	6.20E-07
XKRX	0.00151	0.000328
EGF	0.00106	0.000218
NECTIN4	9.22E-06	9.54E-07
MIR3658	1.07E-05	1.12E-06
CENPI	2.80E-06	2.46E-07
SNORA21	7.90E-08	4.22E-09
EPT1	3.43E-06	3.09E-07
VWDE	0.000169	2.64E-05
GRIN2D	8.53E-05	1.21E-05
RPS24	5.45E-07	3.80E-08
LAD1	1.75E-05	1.98E-06
KIFC1	0.000132	1.99E-05
KRT6A	0.00751	0.00214
CENPL	5.28E-07	3.67E-08
MARVELD3	5.79E-06	5.60E-07
HGD	0.00286	0.000691
NPM3	2.54E-10	6.10E-12
C5orf34	2.15E-11	3.65E-13
DEPTOR	6.79E-08	3.53E-09
QPRT	8.23E-07	6.09E-08
NRK	5.41E-05	7.20E-06
MFAP2	9.37E-07	7.10E-08
SDS	0.00237	0.000556
MALAT1	1.14E-06	8.89E-08
UHMK1	3.46E-06	3.12E-07
PPP2R2C	0.000446	8.09E-05
UBFD1	2.24E-10	5.27E-12
SLCO5A1	2.14E-07	1.32E-08
NKTR	9.52E-07	7.22E-08
MTBP	2.21E-06	1.89E-07
TSPAN5	1.14E-08	4.64E-10
ADAMTS12	1.23E-07	6.98E-09
WASIR2	0.000121	1.80E-05
SALL4	0.000203	3.27E-05
CPD	8.48E-06	8.68E-07
F5	0.00229	0.000534

GNAS	3.62E-08	1.73E-09
DHFR	1.63E-08	6.96E-10
GRTP1	0.000265	4.45E-05
MSTO2P	2.94E-09	9.91E-11
MANEAL	1.20E-05	1.28E-06
RCC1	1.35E-10	2.92E-12
ALDH18A1	2.00E-17	4.38E-20
DBF4	2.46E-12	3.15E-14
FKBP11	5.54E-11	1.07E-12
FUT8-AS1	9.23E-10	2.68E-11
P4HA3	6.07E-07	4.30E-08
KCNN4	2.17E-05	2.53E-06
CDK19	4.15E-15	2.25E-17
MSTO1	2.30E-09	7.50E-11
KNOP1	2.42E-10	5.75E-12
MYO7A	2.85E-09	9.53E-11
FMO1	0.00192	0.000436
MIAT	5.14E-06	4.90E-07
RAB26	2.94E-06	2.60E-07
RFC4	9.93E-11	2.07E-12
BCL2L15	5.05E-05	6.65E-06
GPC6	3.69E-06	3.35E-07
OCIAD2	4.78E-10	1.25E-11
LOC100653	8.31E-07	6.17E-08
HMGA1	2.88E-07	1.83E-08
FEN1	6.86E-09	2.60E-10
LOC101930	2.06E-07	1.26E-08
HIST1H3F	9.25E-05	1.33E-05
PDIA4	2.38E-14	1.62E-16
TDO2	0.00322	0.000795
PAICS	2.26E-12	2.86E-14
MXRA5	6.42E-09	2.40E-10
GYG2	4.08E-06	3.75E-07
PLK4	4.53E-08	2.24E-09
SYT12	0.00022	3.58E-05
HIST1H2BE	6.35E-10	1.74E-11
DNMT3B	1.71E-07	1.02E-08
GINS4	4.77E-07	3.26E-08
LOC102724	3.46E-10	8.63E-12
NUMA1	3.09E-07	1.99E-08
GCLC	0.000663	0.000127
CDC45	0.000116	1.72E-05
ZNF239	1.69E-07	1.01E-08

TLCD1	7.91E-05	1.11E-05
RCN3	5.30E-08	2.67E-09
PLPP5	1.33E-17	2.66E-20
RAD54B	1.46E-05	1.61E-06
KRT80	7.15E-05	9.90E-06
IGDCC4	4.41E-06	4.12E-07
INPP4B	0.000113	1.68E-05
SLC41A2	4.11E-07	2.77E-08
CD27	6.38E-06	6.24E-07
SIX4	8.46E-07	6.31E-08
BLNK	1.84E-11	3.05E-13
ECE2	1.50E-05	1.66E-06
CHGB	0.00575	0.00156
LOC642846	6.66E-09	2.51E-10
PNOC	6.79E-07	4.89E-08
HPSE	1.49E-06	1.20E-07
PPAT	2.72E-11	4.78E-13
PLD5	0.00115	0.00024
DENR	7.14E-13	7.72E-15
RASGEF1A	2.93E-06	2.59E-07
TRIM2	3.31E-07	2.16E-08
FNTB	1.22E-14	7.43E-17
ECT2	9.71E-08	5.36E-09
TDRKH	9.16E-07	6.91E-08
CELSR3	9.91E-07	7.57E-08
CD38	0.000462	8.43E-05
BLM	6.04E-10	1.64E-11
NCAPG2	1.84E-09	5.83E-11
ANP32E	4.52E-08	2.23E-09
ARSE	0.00246	0.000578
PLPP4	7.67E-06	7.74E-07
NME1	3.76E-10	9.51E-12
PTPRF	1.85E-06	1.53E-07
GART	1.35E-10	2.93E-12
ERV3-1	1.12E-07	6.30E-09
MMP7	0.000296	5.05E-05
ESYT2	9.38E-07	7.11E-08
SLC35F2	4.82E-10	1.27E-11
CABYR	2.38E-05	2.82E-06
NBR1	2.89E-14	2.00E-16
EPN3	2.78E-05	3.36E-06
FAM83D	0.000198	3.17E-05
CD19	4.58E-05	5.95E-06

VMP1	6.49E-08	3.36E-09
CEACAM1	1.46E-05	1.61E-06
NRIP3	0.000146	2.23E-05
LINC01207	0.00223	0.000517
CILP2	2.86E-05	3.47E-06
DTL	5.83E-05	7.85E-06
CDT1	3.24E-07	2.11E-08
SAA2-SAA4	0.00519	0.00138
CD72	1.81E-05	2.05E-06
PCDH7	6.63E-05	9.10E-06
SLC6A10PB	0.000291	4.95E-05
SERINC2	2.60E-10	6.27E-12
MCM4	1.00E-09	2.95E-11
LOC100293	2.76E-07	1.76E-08
BIK	4.43E-08	2.18E-09
SEC24A	8.98E-11	1.84E-12
PLOD2	2.77E-13	2.57E-15
EGLN3	2.28E-08	1.02E-09
TIGIT	2.25E-09	7.33E-11
IGLL5	7.74E-06	7.81E-07
PTK2	2.63E-08	1.20E-09
RPS15A	5.78E-09	2.14E-10
SPATS2	1.28E-15	5.71E-18
GAD1	0.000437	7.90E-05
BORA	3.73E-09	1.30E-10
GPX8	3.41E-07	2.23E-08
DOK5	3.59E-07	2.37E-08
SLC28A3	0.00033	5.72E-05
PIM2	4.02E-08	1.95E-09
AGR2	3.60E-07	2.39E-08
BHLHA15	4.26E-07	2.88E-08
PABPC1L	1.10E-10	2.31E-12
UBDGAB	2.34E-05	2.76E-06
FHL2	6.33E-10	1.73E-11
WDHD1	7.93E-09	3.08E-10
MAP7D2	0.00139	3.00E-04
DSC2	1.17E-05	1.25E-06
MB	0.000685	0.000132
CYP27C1	3.79E-05	4.79E-06
HIST2H2BE	6.98E-09	2.66E-10
APOBEC3B	1.89E-06	1.58E-07
NLN	4.20E-09	1.49E-10
TNFSF4	2.23E-10	5.24E-12

UNC5CL	1.28E-07	7.37E-09
DDIT4L	8.45E-05	1.20E-05
PROM2	7.40E-06	7.43E-07
TIMELESS	6.76E-11	1.34E-12
MROH1	1.44E-06	1.16E-07
ZNF117	5.83E-05	7.85E-06
SLAMF7	2.82E-06	2.48E-07
TCTE3	7.22E-12	1.05E-13
CILP	0.000567	0.000106
ALDH3B2	3.25E-05	4.02E-06
LCAL1	0.000618	0.000118
DIO2	2.04E-06	1.72E-07
IGLJ3I	1.54E-05	1.71E-06
FUT3	3.46E-07	2.28E-08
PDGFRL	1.84E-09	5.82E-11
NUP210	3.23E-11	5.78E-13
TMEM45B	7.32E-06	7.33E-07
IGSF9	1.42E-05	1.55E-06
KIF23	5.51E-07	3.85E-08
PDK1	2.61E-15	1.32E-17
MND1	4.50E-06	4.21E-07
HIST1H2BC	8.61E-10	2.48E-11
IGKV1OR2-	9.63E-08	5.30E-09
SRPX2	5.99E-08	3.07E-09
RAD51AP1	2.12E-10	4.94E-12
TRIM59	8.53E-13	9.54E-15
STIL	6.91E-10	1.92E-11
SEL1L2	0.000137	2.09E-05
MYBL2	2.64E-07	1.66E-08
TENM4	4.66E-10	1.22E-11
MATR3	6.80E-09	2.58E-10
PLAU	1.24E-09	3.77E-11
EFNA4	1.42E-10	3.10E-12
CCNA2	2.81E-09	9.38E-11
LOC100131	1.72E-10	3.90E-12
ASPHD2	3.82E-10	9.68E-12
IL2RA	4.31E-07	2.92E-08
IGKV1OR2-	4.71E-08	2.33E-09
CEMIP	1.88E-05	2.15E-06
FGF7	1.64E-06	1.34E-07
HIST1H1C	8.72E-10	2.51E-11
MSI2	6.28E-13	6.61E-15
PI15	0.000186	2.95E-05

PLA2G2D	3.82E-05	4.83E-06
STYK1	5.36E-11	1.02E-12
COL5A1	3.27E-10	8.07E-12
P3H4	2.28E-11	3.91E-13
SEZ6L2	2.18E-07	1.35E-08
THY1	3.33E-08	1.57E-09
PYCR1	7.02E-11	1.40E-12
PAX5	9.68E-06	1.01E-06
DEPDC7	7.30E-07	5.30E-08
SGO2	6.58E-08	3.41E-09
GALNT14	4.43E-05	5.74E-06
ASF1B	1.85E-06	1.54E-07
KNTC1	7.69E-16	3.12E-18
SPRR1B	0.00231	0.000539
IGKC	7.73E-08	4.11E-09
NGEF	3.17E-07	2.05E-08
TK1	5.00E-09	1.80E-10
SPC25	1.40E-06	1.12E-07
TROAP	6.34E-09	2.37E-10
COL5A2	5.54E-13	5.72E-15
IER5L	8.84E-10	2.55E-11
HIST2H2AA	1.31E-12	1.56E-14
ITGA11	6.74E-09	2.55E-10
UGT8	0.00042	7.55E-05
HMGA2	0.00354	0.000887
SERPINB5	0.00312	0.000766
NOX4	6.67E-09	2.52E-10
ZNF280B	2.60E-08	1.19E-09
SRD5A1	1.53E-11	2.44E-13
POLQ	6.66E-08	3.46E-09
PRR11	5.50E-09	2.02E-10
MUC16	0.00165	0.000366
CCNF	1.25E-09	3.80E-11
STK32A	2.24E-06	1.91E-07
GTSE1	3.51E-11	6.36E-13
ESPL1	1.09E-07	6.13E-09
CENPK	2.21E-09	7.18E-11
SFRP2	8.59E-05	1.22E-05
POSTN	1.21E-11	1.87E-13
POLE2	2.18E-08	9.63E-10
KLHDC7B	1.49E-05	1.65E-06
SLC2A1	1.26E-10	2.72E-12
SERPINE2	9.98E-11	2.08E-12

ORC6	2.75E-10	6.66E-12
SAPCD2	8.95E-06	9.22E-07
CDK1	2.72E-09	9.07E-11
CDKN2A	6.04E-06	5.86E-07
LY6K	2.88E-05	3.49E-06
WFDC3	4.95E-05	6.49E-06
LGR4	1.62E-08	6.89E-10
GLB1L3	0.00285	0.000687
CA12	8.12E-07	5.99E-08
TCN1	0.00302	0.000737
ABCC3	4.81E-08	2.39E-09
PLEK2	1.34E-08	5.50E-10
LOC107985	3.38E-10	8.38E-12
HORMAD1	0.00121	0.000254
LINC00665	2.78E-07	1.77E-08
PTGES	1.07E-07	6.00E-09
DNAH14	3.34E-09	1.14E-10
IGLJ3I	4.60E-06	4.32E-07
SFRP4	4.36E-05	5.62E-06
STEAP1	1.86E-09	5.92E-11
PRAME	0.00195	0.000444
S100A2	0.000881	0.000176
MAGEA6	0.00813	0.00235
IGHM	7.17E-08	3.77E-09
SHCBP1	1.54E-09	4.80E-11
CKAP2L	5.71E-06	5.50E-07
RALGPS2	9.08E-16	3.84E-18
CCNE1	4.32E-08	2.12E-09
FOXA1	5.08E-05	6.69E-06
HRASLS	3.31E-05	4.10E-06
PAFAH1B3	2.00E-10	4.62E-12
RHBDL2	9.03E-09	3.56E-10
FUT2	5.39E-08	2.72E-09
CDCA2	2.71E-09	9.04E-11
C1orf106	1.95E-09	6.24E-11
CDCA8	6.72E-07	4.83E-08
CKMT1A	0.000381	6.73E-05
MAGEA6	0.00781	0.00224
CCDC34	5.42E-09	1.98E-10
SLC7A5	3.59E-07	2.38E-08
MMP9	1.26E-07	7.24E-09
IGHG1	8.10E-06	8.25E-07
ATP10B	1.49E-05	1.65E-06

ARHGEF7	1.70E-05	1.92E-06
HOXC6	0.000189	3.02E-05
MCM2	1.97E-10	4.54E-12
SLC7A11	6.73E-06	6.66E-07
PRC1	5.76E-09	2.13E-10
ZBP1	4.79E-07	3.27E-08
MELTF	1.13E-11	1.72E-13
TMEM158	1.98E-05	2.28E-06
HELLS	6.12E-14	4.69E-16
BCL2L11	1.04E-10	2.17E-12
AOC1	0.000319	5.50E-05
HIST1H2AE	8.01E-07	5.90E-08
TOX3	0.000577	0.000109
BMS1P20	4.39E-06	4.09E-07
CCNE2	5.91E-09	2.19E-10
MLIP	4.04E-08	1.96E-09
COL1A2	7.16E-14	5.67E-16
SLC6A8	4.90E-06	4.63E-07
E2F7	2.20E-07	1.36E-08
CPNE5	1.20E-08	4.89E-10
KIF15	5.39E-08	2.72E-09
PTTG1	6.84E-10	1.90E-11
IGLJ3C	1.82E-07	1.09E-08
CKAP2	3.36E-09	1.15E-10
ETV4	4.76E-06	4.49E-07
FER1L4	1.58E-14	1.01E-16
PGM2L1	1.91E-13	1.71E-15
ERO1A	2.04E-11	3.45E-13
ADAM28	2.20E-14	1.45E-16
FANCI	2.65E-11	4.64E-13
UCHL1	0.000166	2.59E-05
HIST1H3F	9.07E-06	9.36E-07
SIX1	0.000284	4.82E-05
KDELR3	2.54E-14	1.74E-16
FA2H	7.76E-08	4.13E-09
LOC100507	3.52E-10	8.79E-12
DERL3	2.96E-11	5.24E-13
MDK	3.49E-07	2.30E-08
HOXB7	1.43E-06	1.14E-07
HMGB3P1	9.56E-10	2.80E-11
C2CD4A	9.63E-06	1.00E-06
IGFBP3	1.32E-12	1.58E-14
FRMD5	1.72E-08	7.39E-10

SPAG5	2.70E-08	1.24E-09
CHEK1	5.85E-11	1.14E-12
GPT2	8.16E-08	4.38E-09
TWIST1	1.60E-07	9.49E-09
TFF1	0.00121	0.000255
COMP	4.99E-05	6.55E-06
IGHV4-31	2.19E-07	1.36E-08
CKAP2	2.49E-07	1.56E-08
IGHV3-23	5.39E-07	3.75E-08
CTTN	8.98E-12	1.34E-13
IGLJ3	6.66E-09	2.51E-10
C15orf48	7.65E-08	4.06E-09
GFRA3	0.000543	0.000101
SLC44A5	5.20E-05	6.87E-06
IGHV4-31	3.80E-07	2.54E-08
AKR1C1	0.00352	0.000882
CDCA3	1.22E-09	3.71E-11
FGL1	0.000119	1.78E-05
CYP27B1	4.34E-08	2.13E-09
ANKRD36BP	4.06E-10	1.04E-11
SPDEF	1.24E-05	1.34E-06
NUSAP1	2.34E-10	5.54E-12
MIR1204	5.37E-13	5.49E-15
NDC80	1.34E-08	5.53E-10
IGLV1-44	8.61E-08	4.66E-09
NMU	0.000149	2.30E-05
ENC1	2.83E-07	1.81E-08
TRIP13	9.77E-05	1.42E-05
C11orf80	9.07E-18	1.58E-20
DEPDC1B	3.48E-09	1.20E-10
MAD2L1	1.33E-09	4.05E-11
IGK	7.26E-08	3.82E-09
NPTX2	5.43E-06	5.20E-07
ATP6V1C2	4.68E-05	6.10E-06
TYMS	1.52E-10	3.36E-12
OIP5	2.16E-06	1.83E-07
AIM2	5.58E-08	2.83E-09
HIST1H2BH	5.10E-12	7.18E-14
CR2	4.80E-06	4.53E-07
AURKB	1.40E-08	5.81E-10
B3GNT3	6.30E-08	3.25E-09
EPHB2	5.32E-07	3.70E-08
MKI67	4.85E-10	1.28E-11

IL4I1	1.01E-09	2.99E-11
GPR87	0.000182	2.89E-05
PLPP2	1.97E-05	2.26E-06
SPOCK1	4.73E-08	2.35E-09
LINC01296	1.63E-11	2.63E-13
BRIP1	4.12E-10	1.06E-11
LOC102725	1.89E-07	1.15E-08
ARNTL2	5.79E-08	2.96E-09
MS4A1	8.13E-05	1.15E-05
IGHD	5.16E-06	4.92E-07
RGS17	2.40E-07	1.50E-08
E2F8	2.93E-09	9.85E-11
RASSF6	1.53E-10	3.40E-12
CEACAM5	0.00461	0.0012
NCAPG	1.43E-10	3.13E-12
CENPU	2.60E-09	8.60E-11
IGLC1	1.95E-06	1.64E-07
MZB1	2.44E-07	1.53E-08
ZWINT	7.38E-11	1.49E-12
CENPE	2.71E-07	1.72E-08
MCM10	1.77E-06	1.46E-07
TRIM59	9.39E-12	1.41E-13
PCP4	4.01E-05	5.12E-06
LOC100293	4.33E-08	2.12E-09
IGHV3-23	3.02E-08	1.40E-09
HJURP	3.29E-08	1.55E-09
KNL1	7.25E-10	2.04E-11
CXCL14	1.90E-05	2.18E-06
LOC102725	1.33E-07	7.68E-09
AKR1B10	0.00217	5.00E-04
HMGB3	7.92E-11	1.61E-12
IGHV4-31	2.36E-08	1.06E-09
IGHV4-31	2.61E-05	3.13E-06
EZH2	1.65E-11	2.67E-13
SNORA11E	1.78E-09	5.62E-11
PCAT6	1.07E-15	4.68E-18
LOC101929	3.03E-10	7.41E-12
IGLJ3C	4.04E-08	1.96E-09
CLDN10	5.10E-06	4.84E-07
HIST1H2BD	5.68E-16	2.18E-18
TNFRSF17	7.91E-07	5.80E-08
LOC105379	2.28E-09	7.42E-11
POU2AF1	1.20E-07	6.82E-09

THBS2	2.39E-14	1.64E-16
CKAP2	1.34E-08	5.53E-10
AFAP1-AS1	0.000173	2.72E-05
CDCA5	4.80E-09	1.72E-10
UHRF1	2.49E-13	2.29E-15
LINC01296	1.09E-10	2.30E-12
FAP	4.87E-11	9.15E-13
GINS2	3.96E-10	1.01E-11
CD79A	1.65E-07	9.81E-09
ANKRD22	1.18E-09	3.55E-11
CDH3	2.02E-08	8.85E-10
TMEM184A	1.16E-09	3.48E-11
GOLM1	6.81E-16	2.70E-18
PRSS2	5.26E-05	6.96E-06
FERMT1	6.47E-10	1.79E-11
RMI2	1.51E-12	1.83E-14
IGHV3-23	2.87E-08	1.33E-09
IGHM	2.51E-08	1.14E-09
WISP1	7.06E-10	1.97E-11
IGHV3-23	2.31E-08	1.03E-09
FAM30A	3.23E-09	1.10E-10
IGKIGK	2.44E-09	8.02E-11
KIF14	1.07E-10	2.25E-12
CDC6	1.20E-09	3.63E-11
FNDC1	1.19E-09	3.57E-11
KIF11	6.00E-11	1.18E-12
FGB	0.00164	0.000363
MMP11	7.65E-10	2.17E-11
KIF20A	7.00E-09	2.67E-10
IGHV4-31	2.33E-08	1.04E-09
KISS1R	9.65E-10	2.83E-11
CCNB2	4.41E-11	8.20E-13
LINC00673	1.09E-08	4.38E-10
LRRC15	1.92E-08	8.36E-10
DNAJC12	7.54E-10	2.14E-11
IGHM	3.68E-08	1.76E-09
KIAA0101	2.04E-11	3.44E-13
KIF18B	9.02E-11	1.85E-12
AURKA	4.63E-12	6.46E-14
IQGAP3	1.53E-10	3.38E-12
NUF2	9.93E-10	2.92E-11
TFAP2A	8.39E-07	6.25E-08
DEPDC1	6.88E-08	3.59E-09

SLC12A8	7.10E-16	2.86E-18
BUB1B	7.52E-12	1.10E-13
FOXM1	2.84E-08	1.31E-09
TTK	5.76E-09	2.12E-10
PBK	1.84E-07	1.11E-08
GPX2	0.00107	0.000221
METTL7B	3.75E-08	1.80E-09
CENPF	7.30E-09	2.80E-10
BUB1	5.96E-11	1.17E-12
CEP55	2.09E-10	4.87E-12
IGHV4-31	6.07E-07	4.30E-08
XDH	7.26E-08	3.82E-09
KIF2C	8.61E-10	2.47E-11
FDCSP	5.07E-07	3.51E-08
LGSN	7.06E-06	7.02E-07
HMMR	2.43E-10	5.79E-12
CDKN3	1.73E-09	5.43E-11
CYP24A1	1.22E-07	6.91E-09
BIRC5	1.32E-08	5.44E-10
SLC35F6	4.98E-08	2.49E-09
DUXAP10	9.94E-13	1.13E-14
COL3A1	7.03E-14	5.51E-16
GINS1	1.13E-13	9.36E-16
AGMAT	4.47E-13	4.44E-15
HS6ST2	7.43E-09	2.86E-10
CT83	2.40E-05	2.85E-06
CCNB1	6.84E-10	1.90E-11
SULF1	2.69E-20	1.18E-23
UBE2C	6.32E-12	9.18E-14
FBXO32	2.38E-14	1.62E-16
KIF4A	2.48E-09	8.14E-11
CRABP2	9.19E-09	3.62E-10
COCH	1.55E-10	3.44E-12
DLGAP5	1.91E-10	4.38E-12
PSAT1	1.41E-13	1.23E-15
IGF2BP3	1.69E-06	1.39E-07
FAM83A	3.06E-08	1.42E-09
MELK	1.29E-14	7.96E-17
SCG5	8.37E-11	1.71E-12
COL1A1	7.16E-14	5.66E-16
CDCA7	4.11E-13	4.02E-15
ANLN	1.49E-10	3.27E-12
SLC2A5	1.88E-15	9.19E-18

UBE2T	1.52E-11	2.43E-13
GCNT3	1.92E-08	8.36E-10
NEK2	6.63E-10	1.83E-11
ADAMDEC1	8.46E-08	4.56E-09
KIF26B	1.38E-17	2.77E-20
TPX2	4.51E-10	1.17E-11
ASPM	3.68E-11	6.73E-13
GJB2	1.94E-12	2.42E-14
RRM2	5.07E-11	9.58E-13
CP	3.63E-09	1.26E-10
SPAG4	1.10E-17	2.07E-20
HIST1H2BJ	3.34E-10	8.28E-12
FCRL5	4.17E-12	5.73E-14
CTHRC1	3.97E-18	4.94E-21
TOP2A	1.63E-11	2.63E-13
CDC20	5.01E-09	1.81E-10
TMPRSS4	1.42E-08	5.93E-10
SPINK1	1.72E-07	1.03E-08
MMP1	4.24E-09	1.51E-10
ADAM12	7.22E-18	1.19E-20
MMP12	2.49E-10	5.97E-12
SPP1	2.70E-17	6.33E-20
COL10A1	4.20E-18	5.44E-21
CXCL13	4.35E-14	3.17E-16
GREM1	2.03E-18	2.12E-21
COL11A1	2.69E-20	1.06E-23

Supplementary Table 2. Differentially expressed miRNA

miRNA	adj.P.Val	p-value
hsa-let-7i	1.70E-05	9.52E-07
hsa-miR-1	8.44E-03	1.94E-03
hsa-miR-106a蛆miR-17	1.42E-02	4.41E-03
hsa-miR-106b	7.52E-05	5.60E-06
hsa-miR-126	1.15E-07	1.42E-09
hsa-miR-1260	1.29E-06	4.02E-08
hsa-miR-1274a	1.71E-06	6.37E-08
hsa-miR-1274b	1.40E-04	1.22E-05
hsa-miR-128	2.86E-03	4.62E-04
hsa-miR-135b	2.43E-04	2.86E-05
hsa-miR-141	1.40E-04	1.15E-05
hsa-miR-142-3p	9.17E-03	2.33E-03

hsa-miR-142-5p	1.48E-04	1.38E-05
hsa-miR-143	1.22E-02	3.50E-03
hsa-miR-145	2.13E-04	2.38E-05
hsa-miR-146a	3.98E-03	7.37E-04
hsa-miR-148a	1.72E-03	2.56E-04
hsa-miR-152	1.38E-02	4.13E-03
hsa-miR-155	3.69E-03	6.42E-04
hsa-miR-183	4.78E-05	2.97E-06
hsa-miR-18a	8.44E-03	1.89E-03
hsa-miR-1915	1.40E-02	4.26E-03
hsa-miR-193b	1.53E-04	1.52E-05
hsa-miR-1975	3.06E-03	5.14E-04
hsa-miR-1979	6.86E-05	4.69E-06
hsa-miR-199a-3p	3.98E-03	7.41E-04
hsa-miR-19a	7.77E-03	1.68E-03
hsa-miR-19b	2.92E-04	3.63E-05
hsa-miR-200a	9.54E-03	2.55E-03
hsa-miR-200b	7.75E-03	1.54E-03
hsa-miR-200c	4.90E-04	6.70E-05
hsa-miR-20a	1.05E-02	2.95E-03
hsa-miR-21	2.75E-08	1.71E-10
hsa-miR-218	2.54E-03	3.94E-04
hsa-miR-25	1.68E-02	5.54E-03
hsa-miR-29a	6.31E-03	1.21E-03
hsa-miR-29b	3.48E-04	4.54E-05
hsa-miR-301a	1.68E-02	5.54E-03
hsa-miR-302e	1.49E-03	2.13E-04
hsa-miR-30a	4.66E-07	8.68E-09
hsa-miR-30b	1.53E-04	1.61E-05
hsa-miR-30d	3.97E-06	1.75E-07
hsa-miR-30e	2.68E-02	9.67E-03
hsa-miR-324-5p	2.38E-02	8.14E-03
hsa-miR-340	2.43E-02	8.46E-03
hsa-miR-345	1.50E-02	4.76E-03
hsa-miR-34a	8.53E-03	2.02E-03
hsa-miR-365	8.53E-03	2.07E-03
hsa-miR-376c	1.71E-02	5.72E-03
hsa-miR-425	7.77E-03	1.69E-03
hsa-miR-429	2.46E-02	8.70E-03
hsa-miR-451	3.97E-06	1.97E-07
hsa-miR-484	8.60E-03	2.14E-03
hsa-miR-500	7.77E-03	1.68E-03
hsa-miR-574-5p	1.38E-02	4.05E-03

hsa-miR-720	9.54E-03	2.53E-03
hsa-miR-93	1.05E-02	2.89E-03
hsa-miR-96	1.03E-06	2.57E-08
