# Supplementary material for: Identification of novel potential drugs and miRNAs biomarkers in lung cancer based on gene co-expression network analysis
Source: Genomics Inform. 2023 Sep 27;21(3):e38. doi: 10.5808/gi.23039 (PMC10584645; doi:10.5808/gi.23039)
Supplement: Supplementary Table 2. — Differentially expressed miRNA [file gi-23039-Supplementary-Table-2.pdf]

Table S2. Differentially expressed miRNA

miRNA	adj.P.Val	P.Value
hsa-let-7i	1.70E-05	9.52E-07
hsa-miR-1	8.44E-03	1.94E-03
hsa-miR-106a蛆miR-17	1.42E-02	4.41E-03
hsa-miR-106b	7.52E-05	5.60E-06
hsa-miR-126	1.15E-07	1.42E-09
hsa-miR-1260	1.29E-06	4.02E-08
hsa-miR-1274a	1.71E-06	6.37E-08
hsa-miR-1274b	1.40E-04	1.22E-05
hsa-miR-128	2.86E-03	4.62E-04
hsa-miR-135b	2.43E-04	2.86E-05
hsa-miR-141	1.40E-04	1.15E-05
hsa-miR-142-3p	9.17E-03	2.33E-03
hsa-miR-142-5p	1.48E-04	1.38E-05
hsa-miR-143	1.22E-02	3.50E-03
hsa-miR-145	2.13E-04	2.38E-05
hsa-miR-146a	3.98E-03	7.37E-04
hsa-miR-148a	1.72E-03	2.56E-04
hsa-miR-152	1.38E-02	4.13E-03
hsa-miR-155	3.69E-03	6.42E-04
hsa-miR-183	4.78E-05	2.97E-06
hsa-miR-18a	8.44E-03	1.89E-03
hsa-miR-1915	1.40E-02	4.26E-03
hsa-miR-193b	1.53E-04	1.52E-05
hsa-miR-1975	3.06E-03	5.14E-04
hsa-miR-1979	6.86E-05	4.69E-06
hsa-miR-199a-3p	3.98E-03	7.41E-04
hsa-miR-19a	7.77E-03	1.68E-03
hsa-miR-19b	2.92E-04	3.63E-05
hsa-miR-200a	9.54E-03	2.55E-03
hsa-miR-200b	7.75E-03	1.54E-03
hsa-miR-200c	4.90E-04	6.70E-05
hsa-miR-20a	1.05E-02	2.95E-03
hsa-miR-21	2.75E-08	1.71E-10
hsa-miR-218	2.54E-03	3.94E-04
hsa-miR-25	1.68E-02	5.54E-03
hsa-miR-29a	6.31E-03	1.21E-03
hsa-miR-29b	3.48E-04	4.54E-05
hsa-miR-301a	1.68E-02	5.54E-03
hsa-miR-302e	1.49E-03	2.13E-04
hsa-miR-30a	4.66E-07	8.68E-09

hsa-miR-30b	1.53E-04	1.61E-05
hsa-miR-30d	3.97E-06	1.75E-07
hsa-miR-30e	2.68E-02	9.67E-03
hsa-miR-324-5p	2.38E-02	8.14E-03
hsa-miR-340	2.43E-02	8.46E-03
hsa-miR-345	1.50E-02	4.76E-03
hsa-miR-34a	8.53E-03	2.02E-03
hsa-miR-365	8.53E-03	2.07E-03
hsa-miR-376c	1.71E-02	5.72E-03
hsa-miR-425	7.77E-03	1.69E-03
hsa-miR-429	2.46E-02	8.70E-03
hsa-miR-451	3.97E-06	1.97E-07
hsa-miR-484	8.60E-03	2.14E-03
hsa-miR-500	7.77E-03	1.68E-03
hsa-miR-574-5p	1.38E-02	4.05E-03
hsa-miR-720	9.54E-03	2.53E-03
hsa-miR-93	1.05E-02	2.89E-03
hsa-miR-96	1.03E-06	2.57E-08
