# Supplementary material for: Identification of novel potential drugs and miRNAs biomarkers in lung cancer based on gene co-expression network analysis
Source: Genomics Inform. 2023 Sep 27;21(3):e38. doi: 10.5808/gi.23039 (PMC10584645; doi:10.5808/gi.23039)
Supplement: Supplementary Table 3. — More information about all modules [file gi-23039-Supplementary-Table-3.pdf]

Table S3. More information about all modules

Module color	Number of genes in module
black	116
darkred	47
Magenta	114
Saddlebrown	33
White	35
Blue	223
Darkturquoise	41
Midnightblue	71
Salmon	84
Yellow	204
Brown	217
Green	123
Orange	38
Sienna3	26
Yellowgreen	24
Cyan	71
Greenyellow	100
Paleturquoise	29
Skyblue	35
Darkgreen	44
Grey	108
Pink	115
Skyblue3	24
Darkgrey	40
Grey60	66
Pulm1	21
Steelblue	31
Darkmagenta	27
Lightcyan	69
Purple	103
Tan	97
Darkolivegreen	27
Lightgreen	66
Red	122
Turquoise	264
Darkorange	36
Lightyellow	60
Royalblue	55
violet	27
