# Supplementary material for: Identification of novel potential drugs and miRNAs biomarkers in lung cancer based on gene co-expression network analysis
Source: Genomics Inform. 2023 Sep 27;21(3):e38. doi: 10.5808/gi.23039 (PMC10584645; doi:10.5808/gi.23039)
Supplement: Supplementary Fig. 1. — Scale independence and mean connectivity of R2 and various soft thresholds. [file gi-23039-Supplementary-Fig-1.pdf]

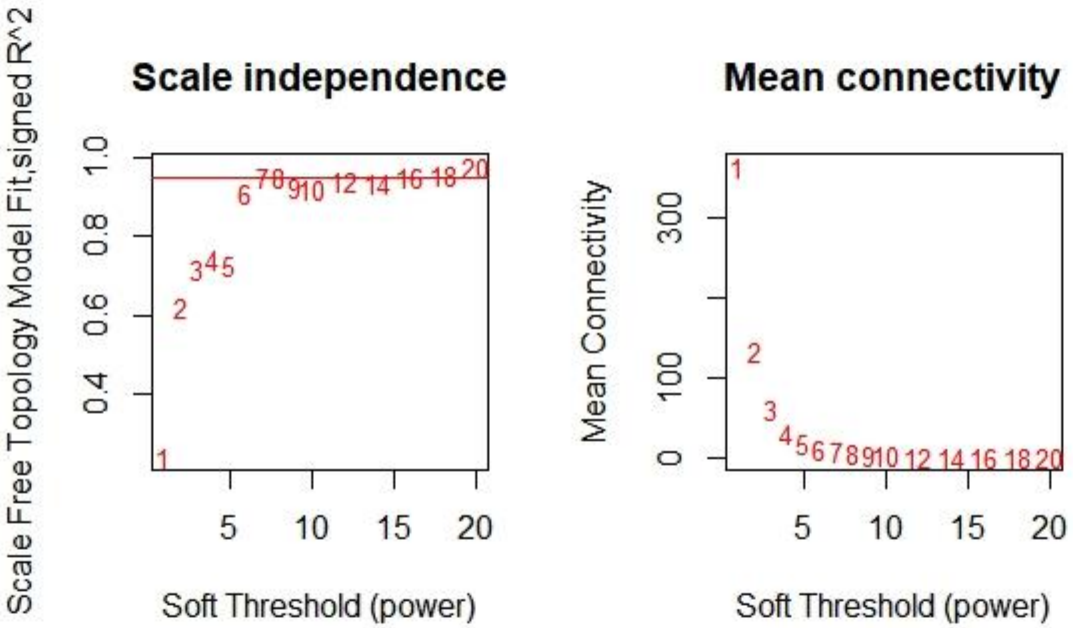


**Supplementary Fig. 1.** Scale independence and mean connectivity of $R^2$ and various soft thresholds.
