# Supplementary figures and images for: Identification of novel potential drugs and miRNAs biomarkers in lung cancer based on gene co-expression network analysis
Source: Genomics Inform. 2023 Sep 27;21(3):e38. doi: 10.5808/gi.23039 (PMC10584645; doi:10.5808/gi.23039)

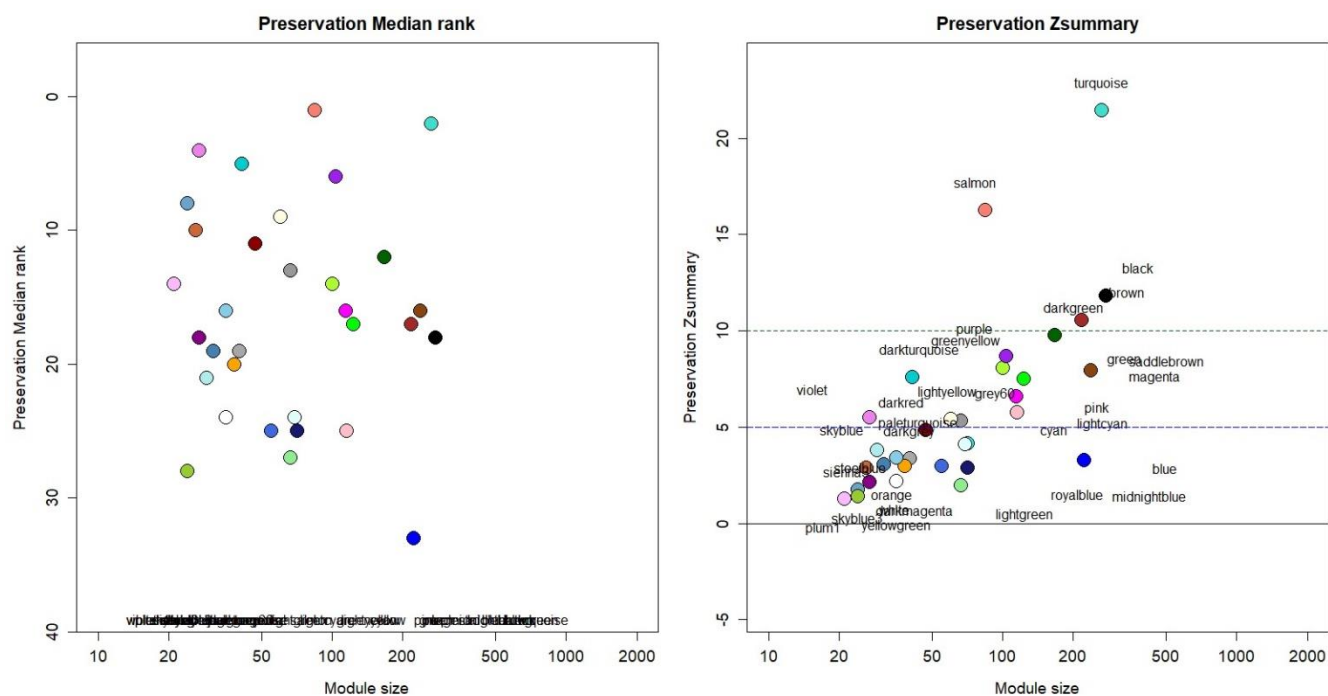

**Supplementary Fig. 2.**  $Z_{\text{summary}}$  score.

Supplement: Supplementary Fig. 2. — Zsummary score. [file gi-23039-Supplementary-Fig-2.pdf]

### Gene dendrogram and module colors

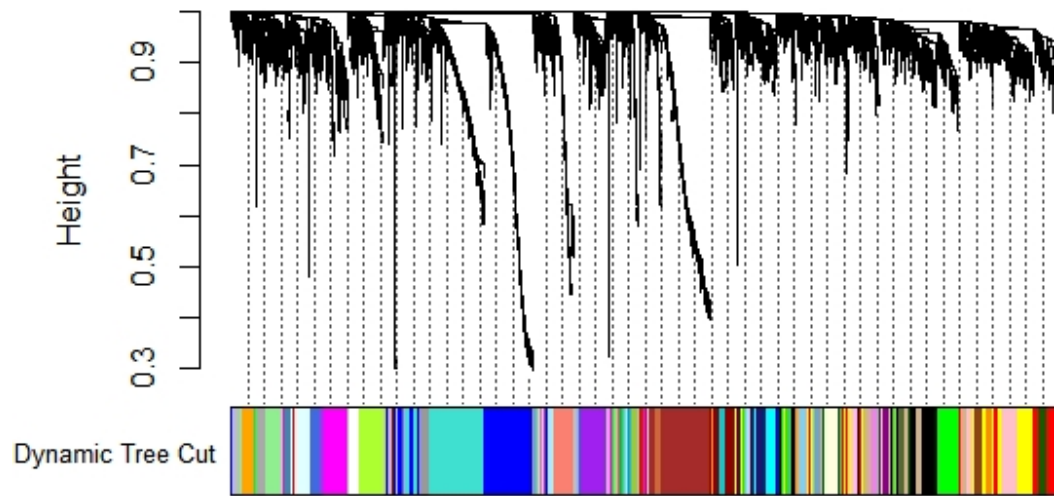

Figure S3. Dendrogram of modules.

Supplement: Supplementary Fig. 3. — Dendrogram of modules. [file gi-23039-Supplementary-Fig-3.pdf]

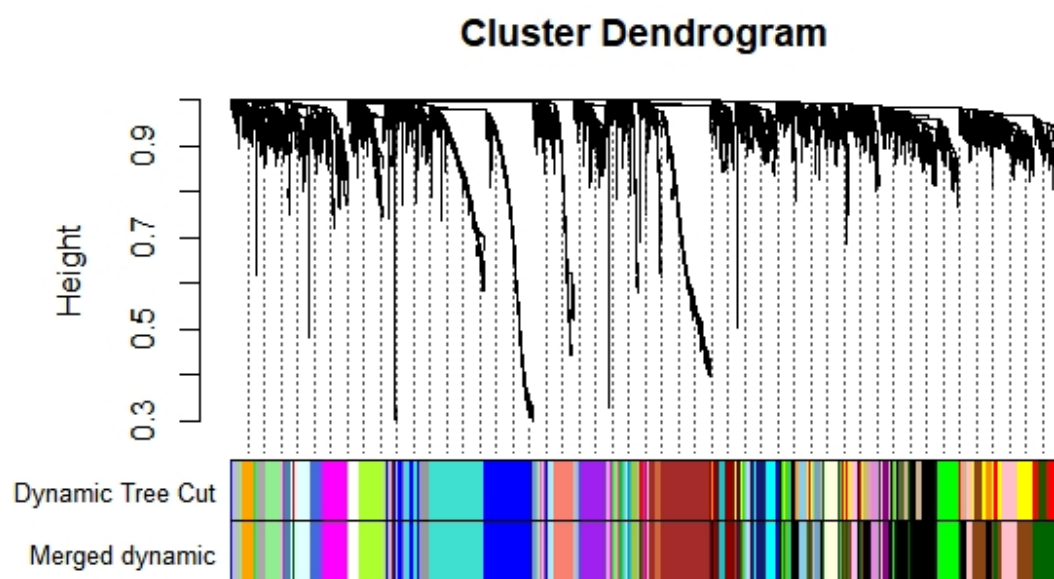

**Figure S4.** Dendrogram after merging modules.

Supplement: Supplementary Fig. 4. — Dendrogram after merging modules. [file gi-23039-Supplementary-Fig-4.pdf]
